# Adaptive Liquid State-Space Networks: A Novel Neuro-Dynamic Architecture for Tabular Classification with Application to Anemia Diagnosis

**DOI:** 10.3390/diagnostics16203276

**Published:** 2026-10-09

**Authors:** Kubilay Muhammed Sunnetci, Faruk Enes Oguz, Ahmet Alkan, Taner Tuncer, Seda Arslan Tuncer

**Affiliations:** 1 Department of Electrical and Electronics Engineering, Osmaniye Korkut Ata University, 80000 Osmaniye, Turkey; kubilaysunnetci@osmaniye.edu.tr; 2 Department of Biomedical Device Technology, Hassa Vocational School, Mustafa Kemal University, 31700 Hatay, Turkey; farukenes.oguz@mku.edu.tr; 3 Department of Electrical and Electronics Engineering, Kahramanmaraş Sütçü İmam University, 46100 Kahramanmaraş, Turkey; aalkan@ksu.edu.tr; 4 Institute of Electronics, Lodz University of Technology, 93-590 Lodz, Poland; 5 Department of Computer Engineering, Fırat University, 23119 Elazığ, Turkey; 6 Department of Software Engineering, Fırat University, 23119 Elazığ, Turkey; satuncer@firat.edu.tr

**Keywords:** anemia, adaptive gates, anemia classification, liquid time constant, state-space models, tabular data

## Abstract

**Background/Objectives:** Anemia is a complex hematological disorder with various subtypes, and its accurate diagnosis is of critical importance for clinical decision-making processes. Traditional machine learning algorithms and standard deep learning models often struggle to model the nonlinear and typically imbalanced nature of clinical tabular data, which negatively impacts the classification performance and reliability of diagnostic systems. **Methods:** This study proposed ALSS-Net, a novel deep learning architecture that combines the State-Space Model (SSM), Liquid Time Constant (LTC), and Adaptive Gates mechanisms, with the aim of classifying difficult-to-diagnose types of anemia with high accuracy, robustness, and statistical reliability. **Results:** Experimental results showed that the Adaptive Liquid State-Space Networks (ALSS-Net) architecture achieved an accuracy of 92.97% on the validation set. Furthermore, on the test dataset, this model obtained an accuracy of 89.13% and an F1 score of 0.8513, outperforming five of the six baseline models by a statistically significant margin (McNemar’s test, *p* < 0.001); the difference with respect to the strongest baseline (MLP) did not reach statistical significance. The ablation studies indicated that the proposed dynamic time constant and adaptive gating mechanisms contributed to the separation of the difficult classes, with adaptive gating accounting for the larger part of the improvement. **Conclusions:** The architecture also produced better-calibrated probability outputs than the compared models, attaining the lowest Brier score and Expected Calibration Error, and degraded more gracefully under increasing feature noise. These findings were obtained on a single, relatively small open-access dataset without external clinical validation; ALSS-Net is therefore presented as a candidate framework for the future development of clinical decision support systems rather than as a validated diagnostic tool.

## 1. Introduction

Anemia is defined as a condition in which the red blood cell mass or the hemoglobin carried by these cells falls below the level required to meet the body’s oxygen demand, and it remains the most common hematological disorder worldwide [1]. According to data from the Global Burden of Disease Study 2021, the prevalence of anemia across all age groups was 24.3% in 2021, corresponding to approximately 1.92 billion people, and anemia resulted in 52.0 million years of life lost due to disability, with only back pain and depressive disorders causing more disability than anemia [2]. The distribution of this burden varies significantly by age, gender, and geography; children under five, women, and countries in sub-Saharan Africa and South Asia stand out as the most affected groups [2]. Therefore, the early and accurate detection of anemia is of critical importance not only for individual patient care but also for the planning of public health policies.

In practice, the diagnosis is made by referring to hemoglobin thresholds established according to age, sex, and pregnancy status [1]. However, this threshold merely indicates the presence of anemia and does not explain its underlying cause. To determine the appropriate treatment, the subtype of anemia must be identified. For this purpose, a morphological classification based on red blood cell indices is traditionally used. In the 1930s, Wintrobe proposed a two-axis scheme based on red blood cell volume and hemoglobin concentration; the first axis defines a classification based on cell size (microcytic, normocytic, macrocytic), while the second axis defines a classification based on intracellular hemoglobin concentration (hypochromic, normochromic, hyperchromic) [3]. With the widespread adoption of automated hematology analyzers, the mean corpuscular volume has become the primary component of this scheme that remains in practical use, while the diagnostic value of the mean corpuscular hemoglobin concentration has diminished; in contrast, the red blood cell distribution width (RDW) and reticulocyte-based indices have been incorporated into the evaluation [3]. While the combined assessment of these indices narrows down the differential diagnosis, confirming the diagnosis requires additional laboratory tests, such as markers of iron metabolism, vitamin B12, and folate [4]. These confirmatory tests, such as serum ferritin, are costly and have more limited availability compared to a Complete Blood Count (CBC) [5].

This situation provides strong motivation to enhance the diagnostic potential of CBC parameters. Although the CBC is a routinely ordered, low-cost, and widely accessible test, it can yield findings that overlap extensively across different subtypes of anemia. Indeed, in cases of microcytic anemia, three primary diagnostic possibilities must be evaluated together: iron deficiency anemia, thalassemia, and anemia of chronic disease, and additional indices such as RDW can only narrow this distinction at the probabilistic level [4]. Furthermore, since the CBC includes not only the red blood cell series but also the white blood cell and platelet series, non-anemic hematologic conditions such as leukemia and thrombocytopenia must also be distinguished within the same dataset. This multi-class and imbalanced structure complicates manual evaluation. When combined with increasing patient volume and the inability to access a hematologist under all circumstances, subtype diagnosis may be delayed, thereby highlighting the need for automated decision support systems [6].

Numerous machine learning approaches have been proposed in the literature for the detection and classification of anemia based on CBC data. A significant portion of these studies have framed the problem in terms of a limited number of categories. Pullakhandam and McRoy [5] showed that iron deficiency anemia can be distinguished from CBC data in a two-class setting using a well-defined machine learning approach, eliminating the need for an expensive ferritin test. Vohra et al. [7] classified the severity of anemia into three classes, mild, moderate, and severe, in an outpatient setting. Gómez Gómez et al. [8] performed a four-class classification using supervised learning algorithms that included not only healthy individuals but also the microcytic, normocytic, and macrocytic subtypes; Awaad et al. [9], on the other hand, proposed a framework that classifies four types of anemia by using ontologically enriched features in conjunction with classical and deep classifiers. Kilicarslan et al. [10] developed two hybrid models based on a stacked autoencoder and a convolutional neural network, optimized using a genetic algorithm, and addressed a five-class problem comprising individuals with hemoglobin anemia, iron, B12, and folate deficiencies, as well as those without anemia. Using the same hemogram dataset, Yagmur et al. [11] proposed hybrid models that combine the TreeBagger method with meta-heuristic algorithms for the same five classes.

In these classifications, the morphological and etiological distinction between subtypes of anemia is partially preserved, while non-anemic hematological conditions such as leukemia and thrombocytopenia are generally excluded. The problem of an increased number of classes significantly complicating the task can be observed in the study by Karagül Yıldız et al. [6]; in this study, which addressed 12 types of anemia collectively and was designed for use in primary care settings, the highest performance remained at 85.6%. This finding indicates that high performance values reported with fewer categories in limited-scale CBC datasets are not maintained when subtype resolution increases.

Despite this body of work, three significant limitations stand out. The first and, from a clinical perspective, the most critical of these is that the calibration of the probability outputs generated by the models has been largely overlooked. However, it has been shown that modern deep neural networks, unlike those of a decade ago, are poorly calibrated and tend to produce overconfident predictions as their depth and width increase [12]. It has also been discussed in the literature that a model with calibration error can lead a clinician to a false perception of risk, regardless of how high its discriminatory power may be, and can thereby cause harm to the patient [13]. Evaluating the distinction between models solely based on global accuracy overlooks the fact that models with similar discriminatory power may differ significantly in terms of the reliability of the probabilities they generate. The second limitation is that performance is generally reported only using global metrics, and class-specific performance in rare subtypes, as well as the model’s robustness to noisy measurements, are largely not examined. Third, the vast majority of current approaches rely on classical machine learning algorithms, shallow ensemble methods, or tree-based classifiers, while modern deep learning architectures specific to tabular data are evaluated only to a limited extent.

Tabular data presents a unique set of challenges for deep learning. Unlike image and text data, tabular data lacks a spatial or sequential structure, its features often have heterogeneous scales, and the number of examples is often limited. Therefore, systematic comparisons have shown that decision tree-based ensemble methods outperform deep networks in many tabular problems [14,15]. On the other hand, architectures that treat attributes as tokens have significantly narrowed this gap in recent years. TabNet [16] uses a sequential attention mechanism to select which features to reason over at each decision step, TabTransformer [17] generates contextual embeddings by passing categorical feature embeddings through self-attention layers, while FT-Transformer [18] models interactions between features by converting all continuous and categorical features into embedded tokens. It has also been reported that this token-based architecture enhances robustness against missing and noisy features [17]. Developments in this field are discussed in detail in relevant review papers [19].

As a parallel line of development, SSMs have emerged as an efficient alternative to Transformer architectures in sequence modeling. The structured state-space sequence model S4 [20] can model long-range dependencies significantly more efficiently than previous approaches; Mamba [21], on the other hand, can selectively pass or forget information across the sequence using a selective mechanism that makes state-space parameters a function of the input. This approach has since been extended to tabular data. MambaTab [22] achieved competitive performance with significantly fewer parameters by applying Mamba to end-to-end supervised learning on tabular datasets; Mambular [23] demonstrated that interpreting features as a sequence and passing them through state-space layers can significantly improve performance in tabular learning.

In the literature on continuous-time neural networks, LTC networks provide dynamic adaptability by defining liquid time constants that vary depending on the hidden state [24]. This approach is based on neural ordinary differential equations [25] and has been extended to closed-form continuous-time networks [26] and neural circuit policies [27]. The integration of liquid dynamics with structured state-space models was initiated by Liquid-S4 [28], which incorporates input-dependent state transitions into the S4 parameterization. A more recent study, however, treated the fact that Mamba-based architectures typically carry out state evolution over a single time scale as a constraint and transferred an input-conditional, multi-scale fluid discretization scheme to selective state-space layers [29]. However, these combinations have been developed primarily in the fields of image and sequence modeling and have not been applied to clinical case data using an attribute-indicator framework. These designs provide multiscale adaptability in the context of image restoration by modulating the active decomposition steps of numerous dynamic branches with degradation-aware gate weights [29]. In this study, however, the time constant is generated separately for each feature token by a single controller shared across all features, and the state update is applied along the feature-token axis. Thus, fluid dynamics are adapted to tabular data that lacks a sequential time structure without the need for a highly branched fusion mechanism.

The dataset used in this study has previously been the subject of classification studies. Cakmak and Pacal [30] compared decision tree, random forest, CatBoost, Gradient Boosting, and XGBoost models on the same class set and reported that tree-based ensembles were able to achieve high accuracy values. Airlangga [31] compared stacking and voting ensembles on the same dataset, while Agrawal and Vemuri [32] evaluated classical learning algorithms for the classification of blood disorders. None of these studies addressed probability calibration, stability under measurement noise, or the statistical significance of differences between models. Furthermore, the very low number of test samples in the smallest classes may limit the reliability of performance metrics for these classes. The aim of this study is not to claim superiority in raw accuracy on the dataset in question, but rather to systematically examine how alternatives with similar discriminative power differ in terms of the reliability of their probability outputs and their robustness to noise.

This study proposes an architecture called ALSS-Net to address this gap. In the proposed architecture, each attribute in the tabular data is treated as a separate token; the adaptive parameters used in the state updates are generated from the input through a shared controller, with the state transition coefficient A determined from the discrete step Δ and the adaptive time constant τ, while B and C are obtained as input-dependent gates. The architecture was applied to the problem of classifying anemia subtypes and associated hematological conditions on an open-access CBC dataset. After removing duplicate records, the dataset contains 14 clinical features and 1232 samples; it consists of nine classes, including healthy individuals as well as microcytic, normocytic, and macrocytic subtypes of anemia, along with non-anemic hematological conditions such as leukemia and thrombocytopenia. In the final analytical cohort the class proportions range from 26.22% to 0.89%, and this imbalance is preserved in the training, validation, and test partitions by stratified sampling, reflecting the natural imbalance commonly observed in clinical datasets. The primary objective of this study is not only to achieve high discriminative power under these conditions but also to present an architecture capable of producing better-calibrated probability outputs and greater robustness to measurement noise than alternatives with similar discriminative power. The main contributions of this study can be summarized as follows:

An ALSS-Net architecture is proposed that combines input-dependent adaptive input–output gates (B, C) generated from feature tokens with liquid time-constant-based adaptive state transitions (τ, Δ), specifically adapted to tabular data through a feature-token framework.

The proposed model was compared with six baseline models based on different mathematical assumptions, namely, Logistic Regression, SVM with an RBF kernel, MLP, 1D-CNN, BiLSTM, and Transformer Encoder, using the same data splits, preprocessing procedures, and random seed.

The evaluation was not limited to global metrics; class-specific performance, probability calibration based on reliability diagrams, Brier score and Expected Calibration Error (ECE), and robustness under increasing feature noise were also examined.

The statistical significance of differences between the models was assessed using McNemar’s tests and bootstrap confidence intervals, and it was evaluated whether the observed advantages in calibration and robustness were maintained independently of the statistical significance of differences in global accuracy.

The contributions of the liquid time-constant controller and adaptive gate mechanism to model performance were disentangled through a systematic ablation study.

To make the scope of this contribution explicit, it is useful to separate what is newly proposed here from what is adapted from existing work. The individual building blocks are not new in isolation: the selective, input-dependent generation of state-space parameters follows Mamba [21], the state-dependent time constant follows the LTC formulation [24], the coupling of liquid dynamics with structured state-space layers follows Liquid-S4 [28] and its multi-scale extensions [29], and the treatment of each feature as a token follows TabTransformer [17] and FT-Transformer [18]. The novelty of ALSS-Net lies in three points. First, the liquid time constant τ and the discretization step Δ are generated per feature token by a single controller shared across all features, so that the effective decay A = exp(−Δ/τ) becomes a property of each clinical variable rather than of a position in a temporal sequence. Second, the input and output gates B and C are produced from the same controller output, so that the contribution of a feature to the state and its read-out are modulated jointly with the decay; in MambaTab [22] and Mambular [23] the recurrence operates over feature positions under a single global time scale. Third, this combination is transferred to static clinical tabular data using a single shared controller, without the multi-branch, degradation-conditioned fusion mechanisms developed for the image-restoration setting [29]. The remaining components, namely the token embedding, the gated feed-forward block, layer normalization, and the residual connections, are standard and are used without modification. The ablation study described in Section 2.2.7 is designed to isolate the contribution of the two proposed mechanisms.

The remainder of the paper is organized as follows: Section 2 introduces the clinical dataset and preprocessing steps used in the study; Section 3 presents the mathematical formulation of the proposed ALSS-Net architecture and the experimental evaluation methodology; Section 4 discusses the findings comparatively; and Section 5 summarizes the conclusions and suggestions for future work.

## 2. Materials and Methods

### 2.1. Materials

**Figure 1:**
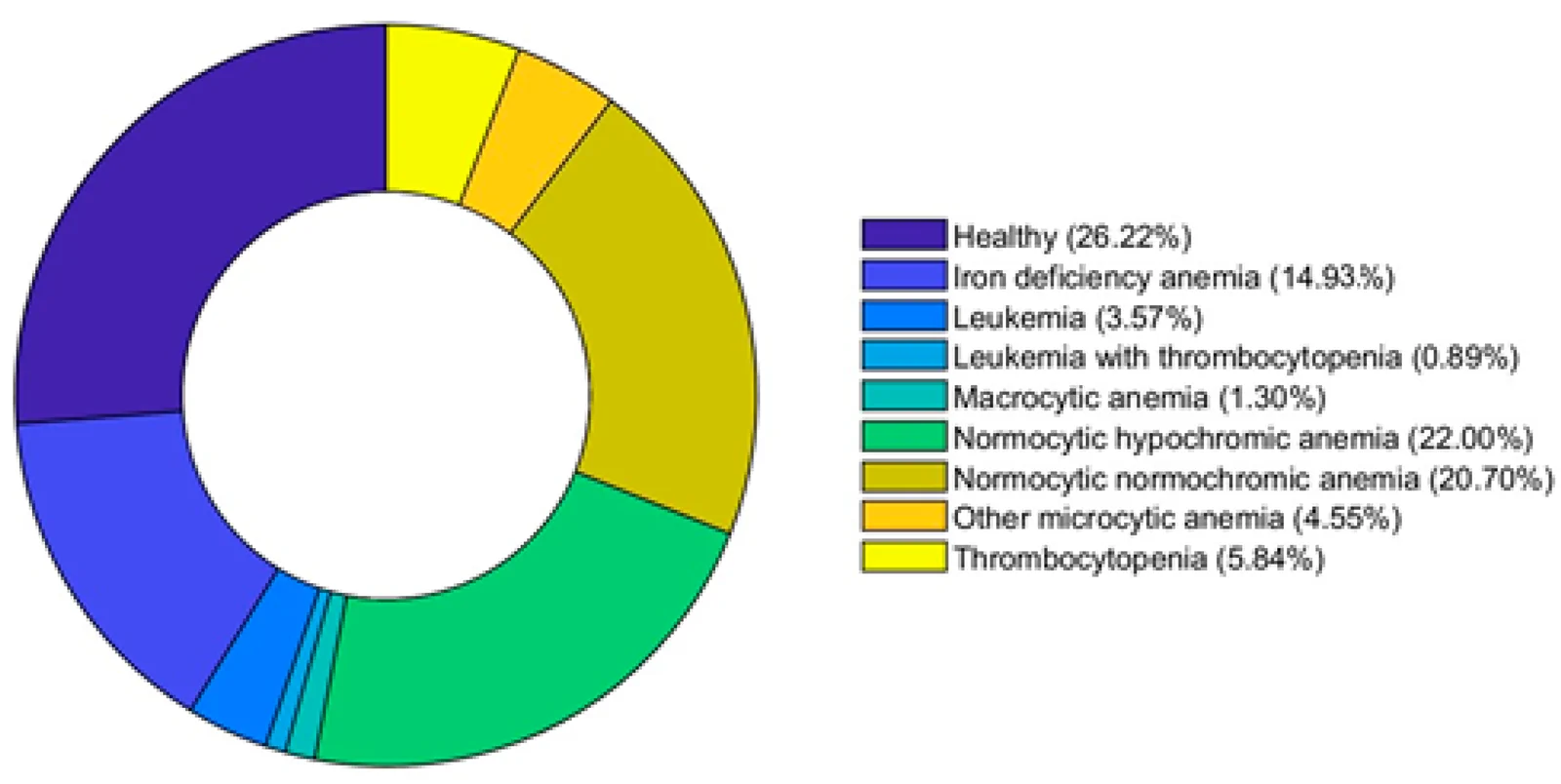
Class distribution and proportional representation of diagnostic categories within the dataset.

This section presents the clinical dataset used in this study to evaluate the classification performance of the proposed ALSS-Net architecture. For the experimental analyses, the “Anemia Types Classification” dataset [33], which is available via open access and can be obtained from the Kaggle platform, was used. This dataset contains clinical parameters obtained from various CBC tests. Its primary purpose is to diagnose anemia based on patients’ blood values and to automatically classify types of anemia. This dataset, which uses manual labels, contains critical features that characterize the blood’s oxygen-carrying capacity, coagulation dynamics, and immune system responses. Among the independent variables that constitute the model’s input features are basic cellular count values such as hemoglobin level (HGB), red blood cell count (RBC), white blood cell count (WBC), platelet count (PLT), and hematocrit (HCT). In addition, the MCV, MCH, and MCHC indices, which measure the mean corpuscular volume, mean corpuscular hemoglobin, and mean corpuscular hemoglobin concentration of red blood cells, are also included. The model also incorporates platelet indices and leukocyte differential parameters, namely plateletcrit (PCT), platelet distribution width (PDW), and neutrophil and lymphocyte values expressed both as percentages and as absolute counts (NEUTp, LYMp, NEUTn, LYMn). PCT is used here in its complete-blood-count sense, denoting the fraction of blood volume occupied by platelets, and not as procalcitonin. The target variable (Diagnosis), on the other hand, consists of categorical labels that determine the patient’s hematological status and the subtype of anemia based on combinations of these 14 different parameters. The developed model aims to achieve high-performance detection by learning the complex clinical relationships among these features through an adaptive state-space mechanism. The overall distribution and structural balance of the classes in the dataset are presented in Figure 1.

Figure 1 presents the distribution and proportional representation of classes in the final analytical cohort of 1232 samples, that is, after the removal of the duplicate records described below. All experiments reported in this manuscript were performed on this cohort. Upon examining the figure, the “healthy” class constitutes the largest group, accounting for 26.22% (*n* = 323). Among the disease classes, normocytic hypochromic anemia (22.00%, *n* = 271) and normocytic normochromic anemia (20.70%, *n* = 255) have the highest data volumes. These major classes are followed by iron deficiency anemia (14.94%, *n* = 184). The remainder of the cohort consists of relatively rarer diagnoses, in the following order: thrombocytopenia (5.84%, *n* = 72), other microcytic anemia (4.55%, *n* = 56), leukemia (3.57%, *n* = 44), macrocytic anemia (1.30%, *n* = 16), and leukemia with thrombocytopenia (0.89%, *n* = 11), which constitutes the smallest group. This distribution, which includes a total of 9 distinct classes, reflects the natural class imbalance frequently encountered in medical data. The importance of the proposed architecture’s ability to distinguish both major classes with high representation and minority classes with representation below 1% was examined in this context.

Because the same open-access dataset has already been used in several classification studies [30,31,32], its provenance warrants an explicit description. The data were released on the Kaggle platform as a collection of routine CBC records, each carrying a single categorical diagnostic label [33]. The labels were assigned manually by the data provider; the diagnostic criteria applied, the qualifications of the annotators, and the confirmatory tests used to establish each label are not documented in the source. No patient-level metadata such as age, sex, comorbidity, medication, or treatment status are distributed with the records, and the records cannot be linked to individual patients. Consequently, no inclusion or exclusion criteria beyond the removal of exactly duplicated rows were applied in this study, since no field is available on which such criteria could be defined. Duplicate detection was performed over the full 14-feature vector together with the label, and 49 records that were identical across all of these fields were removed. These 49 records were distributed across the classes as follows: healthy 13, normocytic normochromic anemia 14, normocytic hypochromic anemia 8, iron deficiency anemia 5, other microcytic anemia 3, leukemia 3, macrocytic anemia 2, thrombocytopenia 1, and leukemia with thrombocytopenia 0, so that the distribution of the dataset as originally distributed, comprising 1281 records, is recoverable from the counts reported here. The ordering of the classes is unchanged by the removal. Near-duplicate records that differ in a single measurement, and repeated measurements originating from the same patient, cannot be identified without patient identifiers and may therefore still be present. These constraints are inherent to the source dataset rather than to the present analysis, and they are revisited among the limitations in Section 5.

Two consequences of this label provenance require explicit statement. First, the fourteen predictors contain no marker of iron metabolism: ferritin, transferrin saturation, serum iron, and total iron-binding capacity are all absent; so, the “iron deficiency anemia” label cannot be checked against the confirmatory biochemistry on which that diagnosis ordinarily rests. The models presented here therefore learn the complete-blood-count signature associated with that label rather than a biochemically confirmed deficiency state. Second, the class names used throughout this manuscript are those assigned in the source dataset, and they are retained unchanged so that the results remain comparable with the studies that have used the same data [30,31,32]; they should not be read as diagnoses established under a clinical reference standard. This applies with particular force to the “leukemia” and “leukemia with thrombocytopenia” categories, since a diagnosis of leukemia requires peripheral blood smear review, blast identification, and immunophenotypic or cytogenetic confirmation, none of which is represented in a fourteen-parameter panel. The corresponding model outputs are to be understood as the recognition of complete-blood-count patterns associated with these labels, and not as a diagnosis of leukemia. The “thrombocytopenia” category is heterogeneous in a comparable way: a reduced platelet count arises from consumptive, immune-mediated, infiltrative, and drug-related processes, among others, and the label as distributed carries no information on which of these applies. The presence of a separate “leukemia with thrombocytopenia” category indicates that at least one combined presentation was assigned its own label rather than being placed in the thrombocytopenia class, but the single-label design of the dataset does not allow co-occurring conditions to be represented in general, and the extent to which the thrombocytopenia class contains isolated rather than accompanied cases cannot be determined from the data as distributed.

Before applying the raw clinical data to the proposed ALSS-Net architecture, various data preprocessing steps were performed to improve data quality and ensure stable model training. In the first stage, the dataset was cleaned of duplicate rows. As a result of this process, 49 duplicate rows were deleted, reducing the total number of examples from 1281 to 1232. Data integrity analyses revealed no missing values, confirming that the dataset was complete. To reliably evaluate the model’s generalization ability, the cleaned dataset was divided into training, validation, and test subsets. The stratified sampling method was used to ensure that the imbalanced distribution between the major and rare anemia classes was preserved at the same ratio across all datasets. Accordingly, the cleaned dataset of 1232 samples was partitioned into 863 training samples (70.0%), 185 validation samples (15.0%), and 184 test samples (14.9%). This 70/15/15 partition is the one used throughout the manuscript, including for the baseline models and the ablation variants, and the corresponding sample counts are the denominators of all validation and test metrics reported in Section 4. Because the partition is stratified, the class proportions are preserved in each subset, and the resulting counts are small for the rarest categories. In the test partition the nine classes are represented by 48 healthy, 41 normocytic hypochromic, 38 normocytic normochromic, 27 iron deficiency, 11 thrombocytopenia, 8 other microcytic, 7 leukemia, 2 macrocytic, and 2 leukemia with thrombocytopenia cases; the corresponding validation counts are 49, 41, 39, 28, 11, 8, 6, 2, and 1. These figures are stated here so that the class-wise scores reported in Section 4 can be read against the number of cases on which each of them rests.

Z-score normalization [34] was used to ensure that the 14 different clinical features had an equal impact on the model and to accelerate the gradient-based optimization process. To maintain methodological validity and prevent data leakage, the mean and standard deviation values required for the standardization process were calculated solely from the training dataset, and the validation and test sets were scaled using these parameters. Finally, the target labels, consisting of 9 different diagnostic categories, were converted into matrix format to ensure the model could operate in accordance with the cross-entropy-based objective function.

### 2.2. The Proposed Method

This section provides a detailed overview of the theoretical foundation, mathematical formulation, model components, and experimental evaluation methodology of the ALSS-Net architecture, which was developed for successful, adaptive, and reliable classification of tabular data. The proposed methodological framework encompasses a multidisciplinary experimental workflow ranging from raw data preprocessing to model training, from the evaluation of comparative baseline models to ablation studies, and from computational efficiency analyses to statistical significance tests. The workflow of these theoretical and experimental steps followed in this study is summarized in Table 1.

The first stage of the methodological workflow involves preparing clinical case data for a reliable learning process. In this context, the raw dataset is cleaned of duplicate cases and divided into training, validation, and test subsets using stratified sampling, while preserving the natural class imbalance in the dataset. To prevent data leakage, Z-score standardization parameters are calculated solely from the training set, and all subsets are scaled using these statistics. Subsequently, each independent variable in the clinical data is treated as a feature token and transformed into a high-dimensional embedded representation space. The LTC controller in the core architecture processes this embedded representation, dynamically generating state-space parameters (A, B, C) specific to each input instead of keeping them fixed, and performs state updates.

To objectively evaluate the classification performance and architectural efficacy of the proposed ALSS-Net, six widely adopted baseline models (Logistic Regression, Support Vector Machine (SVM), Multilayer Perceptron (MLP), 1-Dimensional Convolutional Neural Network (1D-CNN), Bidirectional Long Short-Term Memory (BiLSTM), and Transformer Encoder) were trained and tested under identical experimental conditions and dataset partitions. Furthermore, the evaluation process extended beyond standard global classification metrics. Comprehensive statistical and analytical validation steps were also conducted, including probability calibration analysis via reliability diagrams, robustness assessments through the gradual injection of Gaussian noise, ablation studies to ascertain the contribution of individual model components, and McNemar’s tests to verify the statistical significance of the proposed model’s superiority.

#### 2.2.1. Overview of the Proposed ALSS-Net Architecture

SSMs are mathematical models that define the relationship between inputs and outputs through hidden state variables [20]. The continuous-time linear state-space representation is expressed by the state and output equations given in Equation (1) [20,21]. In this representation, A, B, C, and D denote the model parameters.

(1) $$\dot{h}\left(t\right)=Ah\left(t\right)+Bx\left(t\right),\;y\left(t\right)=Ch\left(t\right)+Dx\left(t\right)$$

In selective SSMs proposed in recent years, certain parameters used in the state update are generated based on the input. Thus, a portion of the model parameters can vary according to the current input. This approach forms the foundation of the selective state update mechanism used particularly in the Mamba architecture [21].

The ALSS-Net proposed in this study combines the classical state-space model with the adaptive time-constant approach used in LTC networks [24]. Recent Liquid-Mamba-based studies have further extended this approach by integrating input-dependent continuous-time dynamics into selective state-space models [21,28,29]. In the proposed architecture, the *A* transition coefficient and the *B* and *C* adaptive gates used in the state update are dynamically determined based on the input features, and this adaptive parameter generation process is described in detail in the following section.

The model developed in this study processes tabular data. Since features in tabular data are mostly represented as independent variables and do not contain an inherently sequential temporal structure, each feature in the input vector is treated as a separate feature token. Thus, a D-dimensional input vector is processed as a feature sequence of length *D*, and the state update operation is applied sequentially to each feature. This approach is consistent with recent token-based deep learning methods proposed for tabular data [17,18].

Because CBC data carry no inherent temporal ordering, it is worth stating explicitly what the sequential state update is intended to accomplish in this setting. The recurrence is not used to represent time. It is used as a mechanism for accumulating evidence across clinical variables: the hidden state h_d_ acts as a running summary of the features processed so far, B_d_ determines how much of the current feature is written into that summary, C_d_ determines how much of the summary is read out at that feature, and A_d_ = exp(−Δ_d_/τ_d_) determines how persistently the information contributed by a feature survives as further features are processed. A large τ_d_ allows a feature to influence the representation of many subsequent features, whereas a small τ_d_ confines its influence locally. This is the mechanism through which a low MCV value can, for example, remain active in the state while MCH, MCHC, and the leukocyte indices are processed, which corresponds to the joint reading a clinician performs when separating microcytic causes of anemia from one another. Since τ, Δ, B, and C are generated from the feature embeddings rather than from position, the parameters attached to a variable follow the variable itself; the ordering of the columns nevertheless remains a design choice, and the global average pooling of Equation (10) is applied over all output tokens so that the final representation does not depend disproportionately on the token processed last.

This section has presented the overall architecture of ALSS-Net, while the following section presents the mathematical formulations governing the input-dependent generation of the embedding layer, LTC controller, *τ* and Δ gates, and the *A*, *B*, and *C* matrices that constitute the adaptive state update mechanism.

#### 2.2.2. Token Embedding and Liquid State-Space Core (Controller, Tau/Delta Gating, B/C Gates)

Each feature xd is transformed into a higher-dimensional embedded representation using a learnable linear transformation (Equation (2)) [17,18]. Thus, the input features are mapped into a shared representation space to be used in the subsequent state update operations.

(2) $$e_{d}=x_{d}W_{d}^{tok}+b_{d}^{tok}$$

The resulting embedded representation *e_d_* is passed to a single controller layer shared across all features (Equation (3)). This layer generates *u_d_*, which is used to compute the adaptive parameters required for the state update.

(3) $$u_{d}=\mathrm{ReLU}\left(W_{ctrl}e_{d}+b_{ctrl}\right)$$

For each feature, the liquid time constant $\tau_{d}$ and the discretization step size $\Delta_{d}$ are computed (Equations (4) and (5)). The $\tau_{d}$ value is kept positive using the softplus activation function and constrained by a predefined minimum value. Similarly, $\Delta_{d}$ is obtained within a predefined range using the sigmoid activation function. These two parameters are generated in an input-dependent manner in accordance with the LTC approach [21,24,29].

(4) $$\tau_{d}=\mathrm{softplus}\left(W_{\tau }u_{d}+b_{\tau }\right)+\tau_{min}$$

(5) $$\Delta_{d}=\sigma \left(W_{\Delta }u_{d}+b_{\Delta }\right)\cdot \Delta_{max}$$

Using the computed *τ_d_* and Δ*_d_* parameters, the state transition coefficient is obtained (Equation (6)) [20,21,29]. This coefficient represents the transition used in the state update equation.

(6) $$A_{d}=\exp\left(-\frac{\Delta_{d}}{\tau_{d}}\right)$$

The input coefficient $B_{d}$ and output coefficient $C_{d}$ are also computed from the same controller output (Equation (7)) [21]. $B_{d}$ controls the extent to which new information is incorporated into the state, whereas $C_{d}$ controls the extent to which the state is reflected in the output. When these gates are kept fixed (ablation variant), the model approaches the behavior of a conventional fixed-parameter SSM.

(7) $$B_{d}=\sigma \left(W_{B}u_{d}+b_{B}\right),\;C_{d}=\tanh\left(W_{C}u_{d}+b_{C}\right)$$

The hidden state is computed using the recurrent state update equation given in Equation (8) [21]. In this update, the previous hidden state and the current feature embedding are combined using the input-dependent $A_{d}$ and $B_{d}$ coefficients. Finally, the output representation is obtained using the $C_{d}$ coefficient in Equation (9) and is combined with the embedded feature representation through a residual connection. This residual connection is used to support information and gradient flow in deep networks [35].

(8) $$h_{d}=A_{d}⊙h_{d-1}+B_{d}⊙e_{d}$$

(9) $$y_{d}=C_{d}⊙h_{d}+e_{d}$$

#### 2.2.3. Feed-Forward Block, Normalization, and Classification Head

All output representations *y_d_* obtained from the state-space block are transformed into a single feature vector *z* via the global average pooling operation given in Equation (10). Thus, a fixed-size representation is obtained for use in subsequent layers.

(10) $$z=\frac{1}{D}\sum_{d}y_{d}$$

The pooled representation is passed through the gated feed-forward block given in Equation (11). In this block, two linear transformations are applied, with one functioning as a gate via the sigmoid activation function. After the outputs of both transformations are multiplied element-wise, the result is combined with the input representation via a residual connection. This structure is designed similarly to Gated Linear Unit (GLU)-based gating mechanisms [36].

(11) $$f=\left(W_{ff1}z+b_{ff1}\right)⊙\sigma \left(W_{ff2}z+b_{ff2}\right)+z$$

The representation obtained from the feed-forward block is normalized via the layer normalization operation given in Equation (12). The normalization process is performed using learnable *γ* and *β* parameters [37].

(12) $$\mathrm{LN}\left(f\right)=\gamma ⊙\frac{f-\mu_{f}}{\sqrt{\sigma_{f}^{2}+\epsilon }}+\beta$$

The normalized feature representation is passed to the final linear classification layer, and the probability values for each class are calculated using the softmax function, as shown in Equation (13). Thus, the model output $\hat{y}$ represents the predicted probability distribution for the classes in the problem.

(13) $$\overset{ˆ}{y}=\mathrm{softmax}\left(W_{cls}\mathrm{LN}\left(f\right)+b_{cls}\right)$$

#### 2.2.4. Baseline Models

This section details comparative baseline models used to comprehensively evaluate the classification performance, generalization capability, and computational efficiency of the proposed ALSS-Net architecture. Six established baseline models, grounded in distinct mathematical paradigms and widely recognized in the literature, were incorporated into the experimental framework to systematically evaluate the contributions of the proposed method in tabular data modeling.

The selected baseline models were structured to correspond to the main algorithmic approaches in the machine learning and deep learning literature. From the statistical model group, Logistic Regression was included in the study as a classical classifier based on linear decision boundaries [38]. SVM with a Radial Basis Function (RBF) kernel was employed to model complex non-linear decision boundaries [39]. Additionally, an MLP model, capable of processing tabular data, was integrated into the study to represent feedforward artificial neural network architectures [40]. 1D-CNNs, which capture local relationships and sequence patterns among features, were also evaluated within this scope [41]. BiLSTM models, which model sequential information flow bidirectionally through their recurrent structure, were selected to demonstrate their spatial and temporal representation capabilities [42]. Finally, the self-attention-based Transformer Encoder architecture, which captures the interdependencies among features, represented the modern attention-based paradigm in the experimental pool [43].

To ensure that the comparison is fair as well as broad, all baseline models were developed under the same protocol as the proposed model. Every model was fitted on the same 863-sample training partition, selected on the same 185-sample validation partition, and evaluated once on the same 184-sample test partition, using z-score statistics computed from the training set alone and a single fixed random seed set at the start of the run. No model, including ALSS-Net, was tuned against the test partition. The four neural baselines (MLP, 1D-CNN, BiLSTM, and Transformer Encoder) were trained with the same optimizer (Adam), the same initial learning rate (1 × 10^−3^), the same mini-batch size (32), and the same early-stopping criterion (patience of 20 epochs monitored on validation loss) as those reported in Table 2 for ALSS-Net. Two settings differ and are stated here explicitly: the neural baselines were given a maximum budget of 50 epochs against 150 for ALSS-Net, and they were trained at a constant learning rate whereas ALSS-Net used a cosine-annealed schedule. All runs were nevertheless governed by the same early-stopping criterion, and the difference in the maximum epoch budget is noted among the limitations in Section 5.

The selected configurations were as follows. Logistic Regression was fitted as a one-vs-all error-correcting output codes ensemble of linear learners with a ridge (L2) penalty at λ = 1 × 10^−4^. The SVM used an RBF kernel within an error-correcting output codes scheme, with a box constraint of 1 and a unit kernel scale, and posterior probabilities obtained through the built-in posterior-fitting procedure. The MLP comprised two hidden layers of 128 and 64 units with ReLU activation and a dropout rate of 0.3 after each. The 1D-CNN comprised two convolutional blocks with 32 and 64 filters of width 5 and same padding, each followed by batch normalization and ReLU, with max pooling of size 2 and stride 2 after the first block, followed by global average pooling, a dense layer of 64 units, and a dropout rate of 0.3. The BiLSTM used 64 hidden units per direction over the feature sequence, with the representation at the final step passed to the classification layer. The Transformer Encoder used a single encoder block with 4 attention heads, a model dimension of 32, and a feed-forward dimension of 64, followed by global average pooling. These configurations were fixed a priori as conventional settings for each model family, and no hyperparameter search was carried out. The comparison is symmetric in this respect: the token dimension, controller dimension, minimum time constant, maximum discretization step, and dropout rate of ALSS-Net reported in Table 2 were likewise fixed a priori and were not searched. The results should therefore be read as a comparison between standard configurations evaluated under a common training and evaluation protocol, rather than as a comparison between individually optimized models; this is stated among the limitations in Section 5.

#### 2.2.5. Training Procedure and Optimization

This section describes the hyperparameter settings used during the training and optimization of the proposed ALSS-Net architecture. All key training parameters used to ensure experimental reproducibility and transparently illustrate the model’s learning dynamics are presented in Table 2. To accelerate the model’s complex matrix operations and optimize its training time, all computational processes were performed using GPU hardware acceleration. During training, categorical cross-entropy loss was combined with an L2 penalty term to maximize performance on multi-class datasets and mitigate overfitting. The model’s weight updates were performed using the Adam optimization algorithm, and the algorithm’s exponential decay rates for the first (*β*_1_) and second (*β*_2_) moment estimates were set to 0.90 and 0.999, respectively. The optimization process began with an initial learning rate of 1 × 10^−3^, and this rate was dynamically adjusted using cosine annealing to enable more precise weight updates as the model’s training progressed.

Under this experimental setup, the training process was executed for a maximum of 150 epochs using a mini-batch size of 32. To prevent overfitting and ensure optimal model convergence, the validation loss was continuously monitored throughout training. An early stopping mechanism was implemented to automatically terminate training if no improvement in validation loss was observed for 20 consecutive epochs (patience = 20).

Additionally, to prevent exploding gradients and maintain training stability, gradient clipping was applied with a global norm threshold of 5.0. L2 weight regularization was set to 1 × 10^−4^, and a dropout rate of 0.20 was incorporated across the network layers to mitigate overfitting. The entire experimental pipeline was initialized with a fixed random seed of 10 to guarantee strict reproducibility.

#### 2.2.6. Evaluation Metrics and Statistical Testing

This section introduces the metrics and visual analysis tools used to evaluate the classification performance, reliability, and statistical significance of the proposed ALSS-Net architecture and the baseline models included in the experimental process from multiple perspectives. Accuracy, precision, recall, specificity, F1-score, and Area Under the Curve (AUC) were employed to measure both overall and class-specific performance on tabular datasets [44,45,46]. To mitigate evaluation bias stemming from imbalanced class distributions typical of clinical datasets, the analysis incorporated fine-grained per-class metrics alongside multi-perspective diagnostic tools [47].

Classifiers’ tendency to make incorrect predictions, misclassification patterns, and the capacity of decision boundaries were examined using graphical evaluation tools. In this context, confusion matrices were constructed to directly compare the actual and predicted label distributions. Receiver Operating Characteristic (ROC) curves were obtained to observe the relationship between models at different decision thresholds, and Precision–Recall curves were generated to determine the performance on rare classes [47,48]. Additionally, probability calibration was evaluated using reliability diagrams, ECE, and Brier score to assess the reliability of the probabilistic outputs and confidence estimates of the models [12,49].

A two-stage statistical evaluation procedure was used in this study to verify whether the performance differences between the models were due to random chance. The McNemar test was applied to examine the pairwise prediction dependencies and error distributions between the proposed ALSS-Net and each baseline model [50]. In this test, which examines the homogeneity of error rates between two models, a *p*-value < 0.05 confirms that the observed performance difference is statistically significant. Additionally, the Bootstrap resampling method was used to determine the stability of the calculated accuracy and F1-score metrics and to provide reliable non-parametric interval estimates beyond simple point metrics [51].

The bootstrap procedure was applied to the 184-sample test partition. In each of B = 1000 replications, a sample of equal size was drawn from the test predictions with replacement and accuracy and macro F1-score were recomputed. The same resampled indices were used for every model within a given replication, so that all models are compared on identical resamples. The intervals reported in Results Section are the 2.5th and 97.5th percentiles of the resulting distributions, corresponding to a confidence level of 95%, and the reported standard deviations are the standard deviations of those distributions. The trained models were not refitted within the resampling loop; so, these intervals quantify the sampling variability of the test partition and not the variability that would arise from repeated training under different initializations.

Because the proposed model is compared with six baseline models, the McNemar tests reported in Results Section constitute a family of pairwise comparisons. The *p*-values given in Results Section are unadjusted. Applying a Holm–Bonferroni correction to the six comparisons involving ALSS-Net does not alter any of the conclusions drawn from them: the five comparisons reported at *p* < 0.001 remain significant at the 5% family-wise error rate, and the comparison with the MLP baseline (*p* = 0.2636) remains non-significant. All McNemar tests were computed on the paired prediction vectors obtained from the identical test partition, which satisfies the pairing requirement of the test.

The robustness analysis reported in Section 4 was carried out on the same test partition. Gaussian noise drawn from a zero-mean distribution was added independently to every feature of every test sample, at noise standard deviations of 0, 0.05, 0.10, 0.20, and 0.30 (Results Section). The perturbation was applied after z-score normalization and was scaled by the per-feature standard deviation of the normalized test data, so that the noise magnitude is expressed in units of each feature’s own dispersion and an equivalent relative perturbation is applied to variables measured on very different clinical scales. The noise was resampled independently for each feature and each sample; no noise was added to the training or validation partitions, and the models were neither retrained nor recalibrated at any noise level. The analysis therefore measures the stability of already-trained decision functions under simulated measurement error rather than the benefit of noise-augmented training.

#### 2.2.7. Ablation Study Design

This section outlines the methodological framework of an ablation study designed to systematically analyze the contributions of the two core components (the liquid time-constant controller and the adaptive input-output gates) constituting the proposed ALSS-Net architecture to classification performance. In this context, it is necessary to reveal the individual impact of these two components on the model’s performance separately. Otherwise, it would remain unclear which mechanism is responsible for the observed performance gains.

In this regard, Table 3 lists various combinations of the LTC controller (*τ*, Δ) and the adaptive gate mechanism (*B*, *C*). When a component is disabled, fixed, static learnable scalars (*τ*_0_, Δ_0_) or unit gates are used instead of input-dependent parameter generation. Thus, all other components of the architecture remain unchanged. The four variants obtained in this manner are as follows: (i) Base SSM, corresponding to a classical state-space model with fixed parameters in which both the LTC and adaptive gates are disabled; (ii) SSM + Liquid Tau, where only the LTC controller is enabled; (iii) SSM + Adaptive Gates, where only the adaptive gate mechanism is enabled; and (iv) ALSS-Net, corresponding to the proposed architecture in which both components are activated together.

Four variants were trained from scratch using the same hyperparameters and training/validation/test splits to ensure methodological validity and comparability. The performance of each variant was evaluated separately on the validation and test sets using accuracy, F1-score, AUC, and confusion matrices. The comparative findings and the contributions of the components to performance are presented in the Results and Discussion section.

#### 2.2.8. Experimental Setup and Implementation Details

This section highlights the hardware infrastructure, software environment, and implementation standards of the proposed ALSS-Net architecture and the baseline models used for comparison. All experimental procedures, model design, optimization cycles, and statistical analyses were carried out in the MATLAB R2026b programming environment. Unlike classical neural networks, the LTC controller and the input-dependent adaptive state-space (*A*, *B*, *C*) gates in the proposed ALSS-Net architecture are not available in standard libraries. Therefore, this architecture was developed and integrated into the system using MATLAB’s dlarray infrastructure, employing a custom training loop approach in which the learnable parameters are stored within a struct and the forward pass was defined as a custom function.

The models were run on a CUDA-enabled NVIDIA GPU to manage computational complexity and enable the parallelization of state-space matrix operations. To ensure the methodological validity of the comparative analyses (SVM, Logistic Regression, MLP, 1D-CNN, BiLSTM, and Transformer), all baseline architectures were run using the same data splits, preprocessing steps, and system random seed (10) as the proposed ALSS-Net. To maximize experimental reproducibility, all model weights, probability calibration diagrams, and prediction sequences generated during the training process were exported and recorded. Thus, potential variations at the hardware and data levels were controlled, ensuring that only the impact of architectural differences on performance was examined within this scope.

## 3. Results

In this section, the experimental performance, probability calibration capabilities, robustness to noise, and statistical significance of the proposed ALSS-Net architecture and six baseline models (SVM RBF, Logistic Regression, MLP, 1D-CNN, BiLSTM, and Transformer Encoder) are evaluated from multiple perspectives. The evaluation process is structured to include training dynamics, quantitative classification metrics, McNemar and Bootstrap-based statistical tests, graphical decision boundary analyses, calibration measures (ECE, Brier score), noise sensitivity analyses, and component-focused ablation studies. The findings indicate that ALSS-Net achieves highly competitive performance across both classification accuracy and reliability, and that the proposed architectural innovations yield robust results consistent with theoretical expectations.

**Figure 2:**
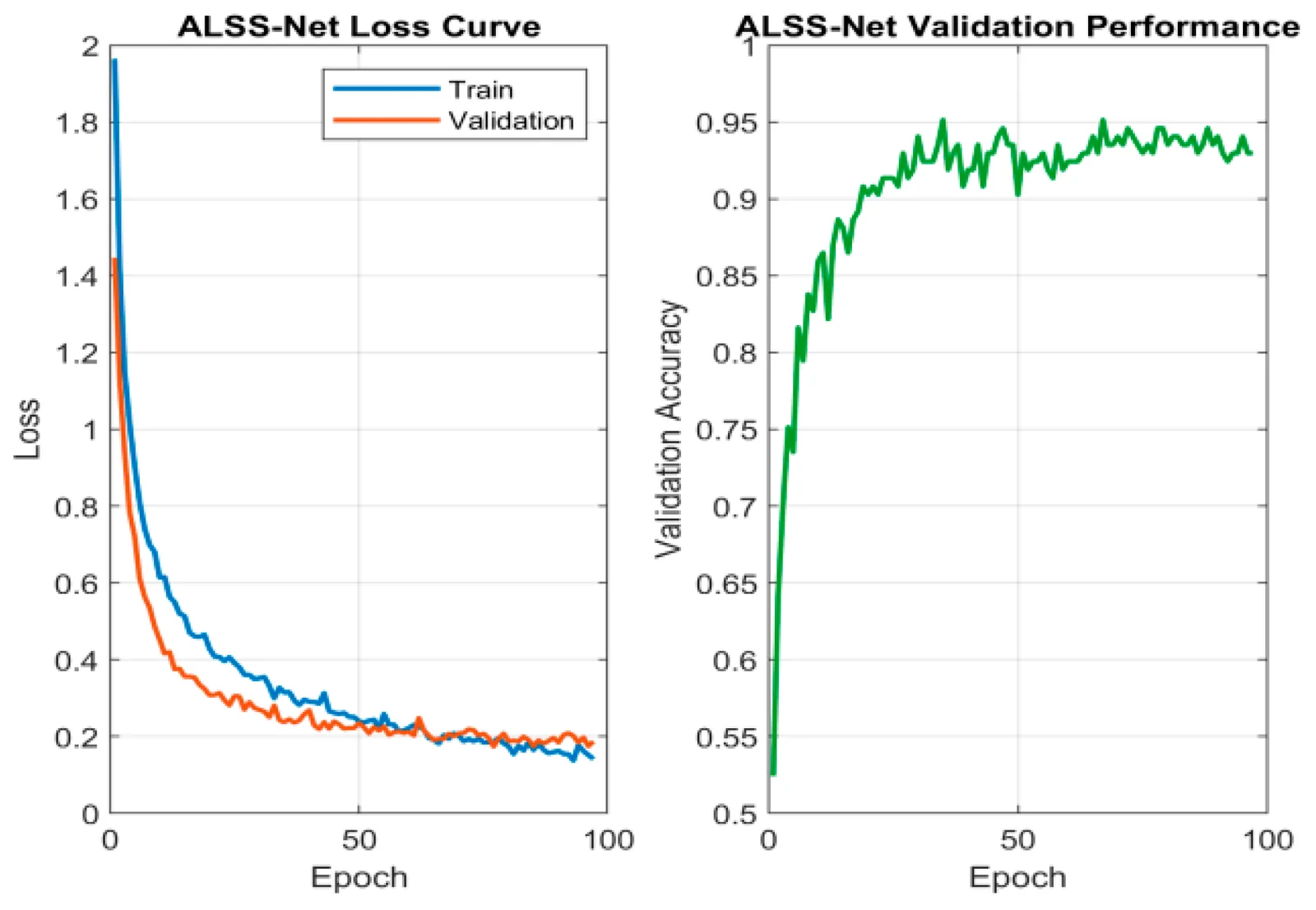
Training loss, validation loss, and validation accuracy dynamics of ALSS-Net across training epochs.

Figure 2 shows the dynamics of the training loss, validation loss, and validation accuracy exhibited by the ALSS-Net architecture over the completed training run. Training was configured for a maximum of 150 epochs with an early-stopping patience of 20 epochs, as reported in Table 2, and the run shown was terminated by the early-stopping criterion after approximately 100 epochs; the horizontal axis of Figure 2 therefore spans the completed run rather than the maximum epoch budget. As observed in the left panel, both training and validation losses exhibited a steep decline during the initial 20 epochs and then converged to a range of approximately 0.15–0.20 as training progressed. The close alignment between the training and validation loss curves throughout all epochs indicated that the model was resilient to overfitting. The validation accuracy curve shown in the right panel indicated that the model rapidly increased from an initial level of ~52.5% to above 90% within the first 20 epochs, and subsequently stabilized within the 92–95% range. These convergence trends confirmed the optimization stability of the ALSS-Net architecture and the effectiveness of gradient flow.

**Figure 3:**
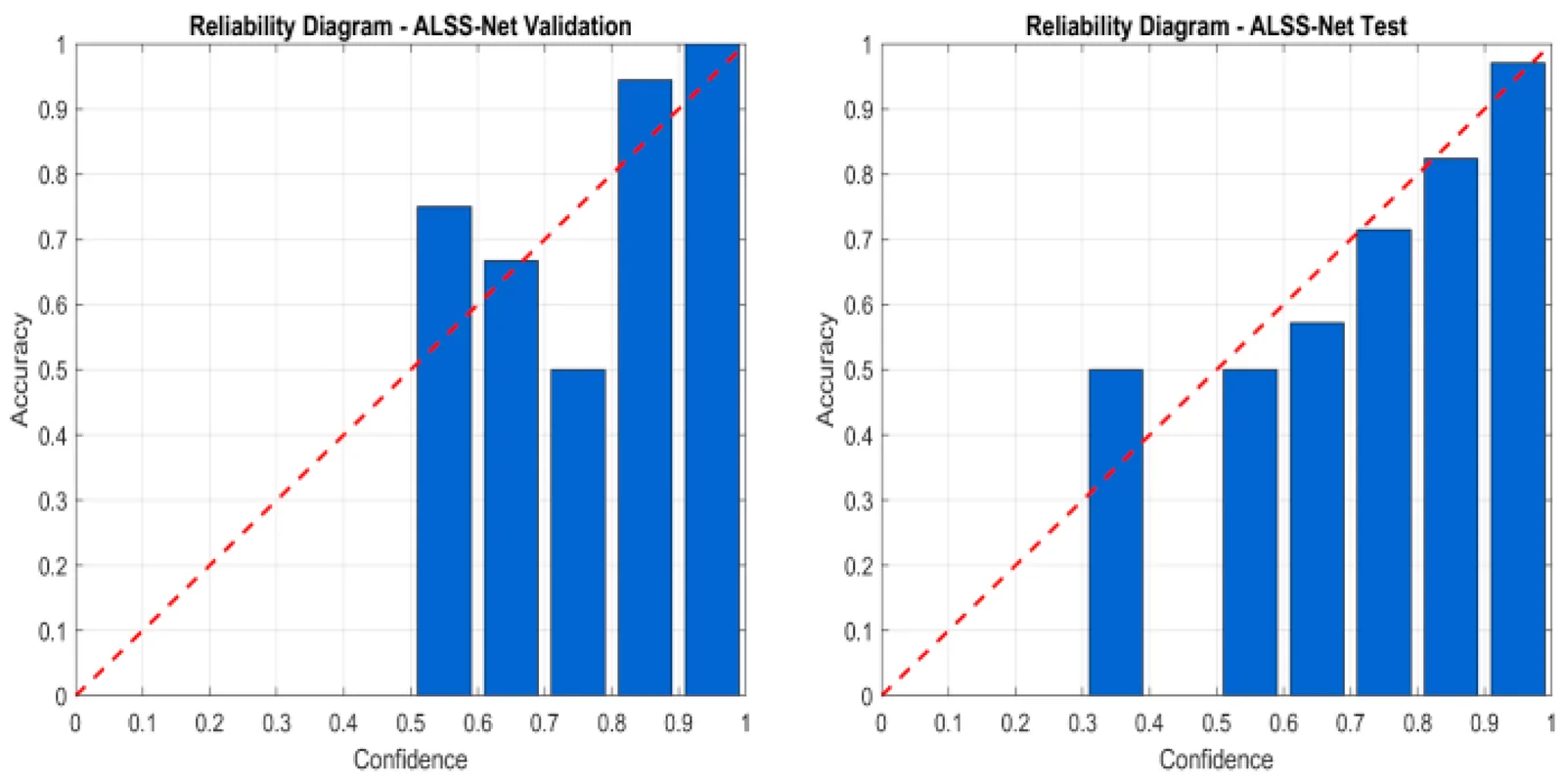
Reliability diagrams evaluating the probability calibration performance of ALSS-Net on validation and test sets.

Figure 3 presents reliability diagrams evaluating the probability calibration performance of the ALSS-Net architecture on the validation and test datasets. In the graphs, the x-axis represents the model’s confidence scores, while the y-axis represents the observed accuracy within the corresponding confidence bins. The red dashed line indicates the ideal calibration reference (*y* = *x*). Across both validation and test sets, the model’s confidence scores aligned well with the ideal calibration line. Particularly on the test set, the close alignment of the bars in the 0.80–1.00 confidence range with the reference line indicated that ALSS-Net successfully mitigated overconfidence (miscalibration), a problem commonly encountered in deep learning models. The balanced distribution observed even at low and medium confidence levels further indicated that the model’s probabilistic outputs were well aligned with the observed predictive performance, suggesting that the probabilistic predictions were well calibrated.

**Figure 4:**
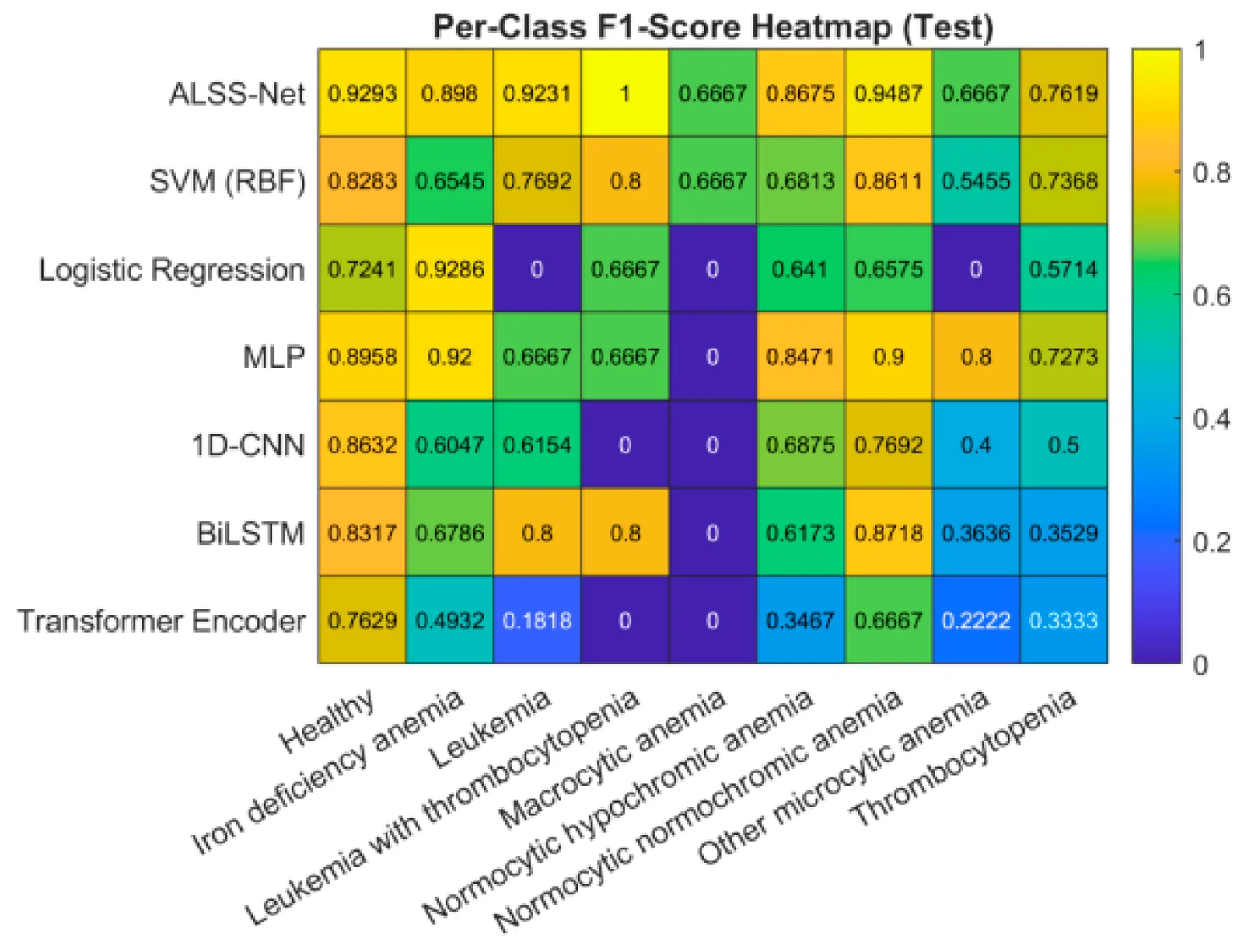
Per-class F1-score heatmap comparing ALSS-Net with baseline models on the test set.

Figure 4 gives a class-based F1-score heatmap detailing the performance of the ALSS-Net architecture and the compared baseline models across the nine classes in the test set. The color scale in the heatmap ranges from 0 (dark blue, lowest performance) to 1 (yellow, perfect performance), visualizing the models’ class-wise performance across the imbalanced dataset and challenging clinical classes. Upon evaluating the heatmap, it is evident that ALSS-Net demonstrated robust performance by achieving balanced F1 scores across all diagnostic categories. In particular, the model achieved an excellent F1 score in the “Leukemia with thrombocytopenia” class and also exhibited high performance in the “Normocytic normochromic anemia” (0.9487), “Healthy” (0.9293), “Leukemia” (0.9231), and “Iron deficiency anemia” (0.8980) classes. An analysis of the ALSS-Net architecture revealed that the absence of any zero or near-zero performance cells in any clinical class suggests that the architecture was able to learn generalizable feature representations even under heterogeneous and imbalanced data distributions. The performance of the baseline models revealed notable bottlenecks in both shallow and deep architectures, particularly in minority classes that were more difficult to classify. For example, in the “Macrocytic Anemia” class, all of the Logistic Regression, MLP, 1D-CNN, BiLSTM, and Transformer Encoder architectures failed completely, yielding an F1 score of 0. Only ALSS-Net and SVM (RBF) were able to classify correctly in this class, achieving an F1 score of 0.6667. Similarly, in the “Leukemia with thrombocytopenia” class, while the 1D-CNN and Transformer Encoder models remained at an F1 score of 0, ALSS-Net correctly distinguished all examples in the class with a very high score. The Transformer Encoder’s poor performance in many classes (such as F1 scores of 0 and 0.1818) indicates that the self-attention mechanism tends to overfit when the dataset size is limited. It was noted that the dynamic architecture used by ALSS-Net was less affected by class imbalances and showed stable performance across all clinically critical subgroups.

These class-wise values must nevertheless be read together with the number of test cases available in each class. Within the 184-sample test partition the rarest categories are represented by very few cases: the “Leukemia with thrombocytopenia” and “Macrocytic anemia” categories contribute two test cases each, and the “Other microcytic anemia” category contributes eight. The F1-score of 1.0000 obtained in the “Leukemia with thrombocytopenia” class therefore reflects the correct assignment of two cases rather than demonstrated generalization to the condition, and the F1-score of 0.6667 obtained by ALSS-Net and SVM (RBF) in the “Macrocytic anemia” class corresponds to one of two cases recovered. A single reassignment in either class would change the score by a large margin, and the F1-score of 0.6667 obtained in the “Other microcytic anemia” class rests on a similarly narrow base. The failure of the remaining baselines to recover any case in these categories remains informative, since it indicates a systematic collapse onto the majority classes, but the corresponding scores achieved by ALSS-Net should be interpreted as evidence that the proposed architecture does not collapse in this way, rather than as an estimate of its accuracy on rare anemia subtypes in clinical practice.

It is also worth situating these results with respect to the simple index-based rules used in routine practice. The best known of these, the Mentzer index, is the ratio of mean corpuscular volume to red blood cell count and is computable from two of the fourteen predictors used here; values below 13 are taken to favour beta-thalassemia trait and values above 13 to favour iron deficiency. Rules of this kind retain considerable value, precisely because they are transparent, require no computational infrastructure, and can be applied at the bench. A direct quantitative comparison is nevertheless not possible on the present dataset. The discrimination that the Mentzer index is designed to perform is between iron deficiency and beta-thalassemia trait, and no thalassemia category appears among the nine labels; so, the index would be evaluated on a distinction it was not constructed to make. The two tasks also differ in scope: the index addresses one binary question within the microcytic group, whereas the models compared here assign one of nine categories spanning microcytic, normocytic, and macrocytic anemias together with platelet and leukocyte abnormalities. A dataset containing a confirmed thalassemia trait category, together with the iron studies required to establish the reference standard, would allow the comparison to be conducted properly, and we regard it as a worthwhile component of the external validation discussed in Section 5.

Table 4 and Table 5 provide the accuracy, precision, recall, F1 score, and AUC values obtained from the validation and test datasets, respectively, for the ALSS-Net architecture and the six baseline models under comparison. Upon examining the validation dataset results, it was observed that ALSS-Net achieved the highest scores across all evaluation metrics, with an accuracy of 92.97%, precision of 0.8104, recall of 0.8051, F1-score of 0.8013, and AUC of 0.9977. In this study, the second-strongest model after ALSS-Net was MLP (88.10% accuracy, 0.7425 F1-score), while SVM (RBF) ranked third with 82.70% accuracy. 1D-CNN (74.59% accuracy), BiLSTM (74.59% accuracy), and Logistic Regression (72.43% accuracy) achieved moderate performance. The Transformer Encoder, on the other hand, performed the worst on the relevant dataset with 54.05% accuracy. In the test dataset results, which revealed the model’s generalization capacity on unseen data, ALSS-Net maintained its lead, achieving an accuracy of 89.13%, a precision of 0.8982, a recall of 0.8288, an F1 score of 0.8513, and an AUC of 0.9931. On the test set, the MLP model achieved a very close and competitive performance with 85.86% accuracy and an F1 score of 0.7137. ALSS-Net, particularly with its F1-score of 0.8513, showed that it provided a more balanced classification against class imbalances and achieved the highest average performance scores across all metrics.

Table 6 presents the results of the Bootstrap resampling performed to evaluate the estimation stability and variance sensitivity of the models’ accuracy and F1-score metrics on the test set. Upon examining the obtained Bootstrap distributions, it was observed that ALSS-Net achieved the highest 95% accuracy confidence interval, ranging from 84.78% to 93.47%, and the lowest standard deviation among all models, at 0.0227. This low standard deviation and high lower bound confirm that ALSS-Net provides more robust estimation stability against potential sampling fluctuations in the dataset. The closest competitor, the MLP model, followed ALSS-Net’s confidence limits with an accuracy confidence interval of [80.43%, 90.76%] and a standard deviation of 0.0264, demonstrating its stability. In the F1-score bootstrap distributions, ALSS-Net was also the only model capable of exceeding the 0.90 upper threshold. This model positioned its overall distribution band at higher levels compared to the MLP and SVM (RBF) models. Architectures such as 1D-CNN, BiLSTM, and Transformer Encoder, on the other hand, exhibited relatively lower confidence intervals and wider fluctuation ranges. The analysis reveals that ALSS-Net’s performance is not a result of random chance but is consistently maintained across different resampled test subsets.

**Figure 5:**
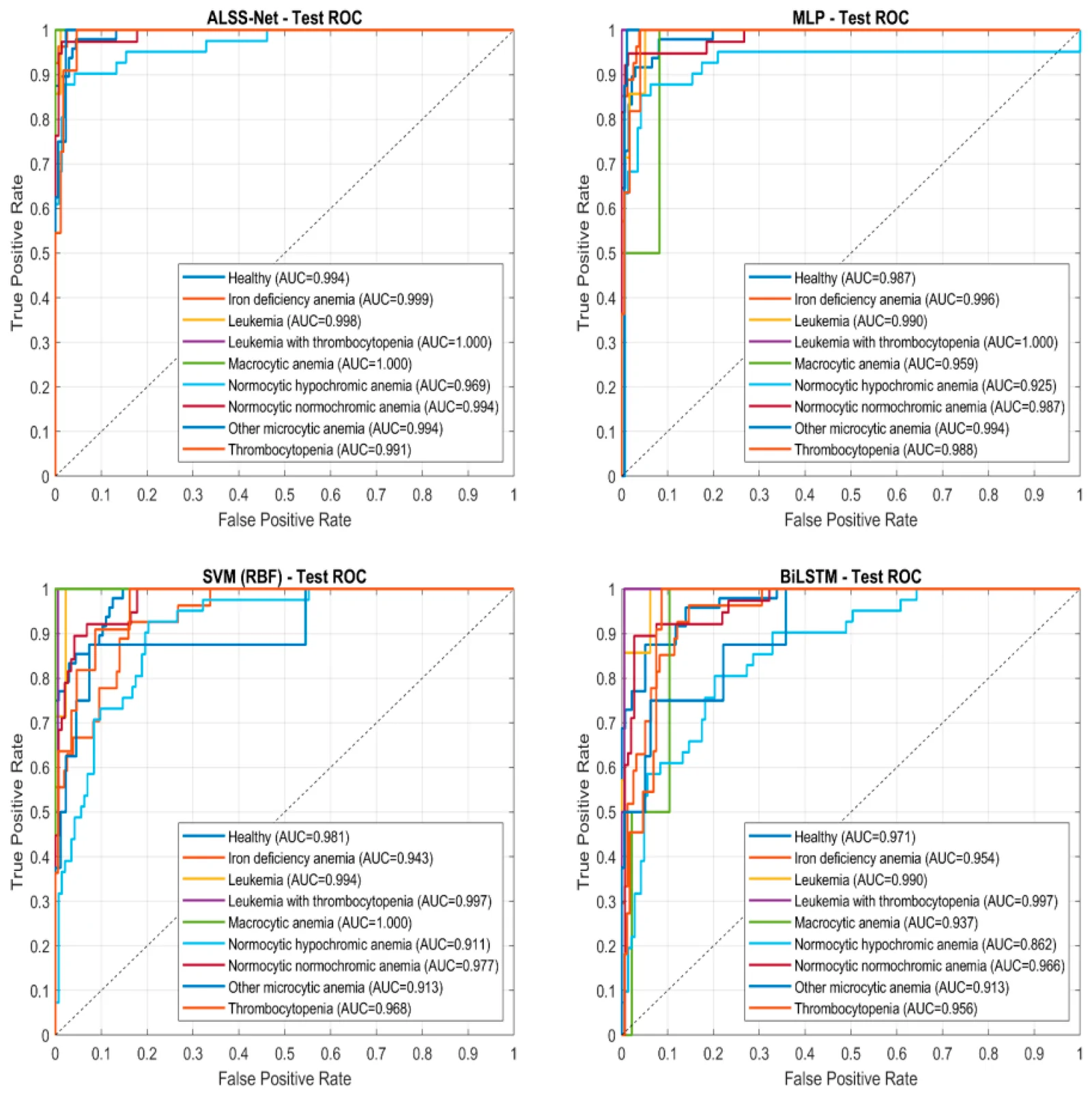
ROC curves and class-wise AUC values for selected baseline models and ALSS-Net on the test set.

**Figure 6:**
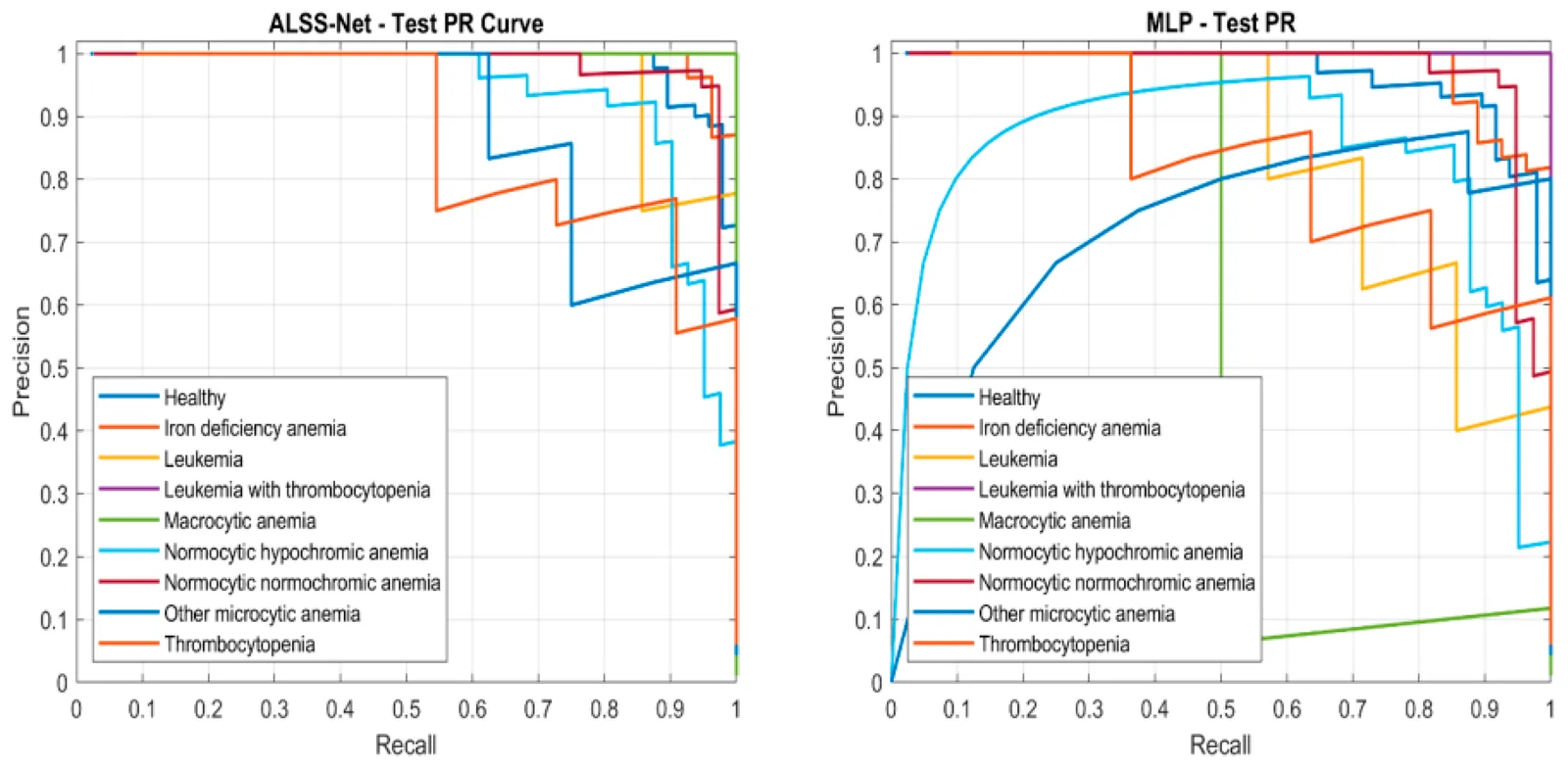

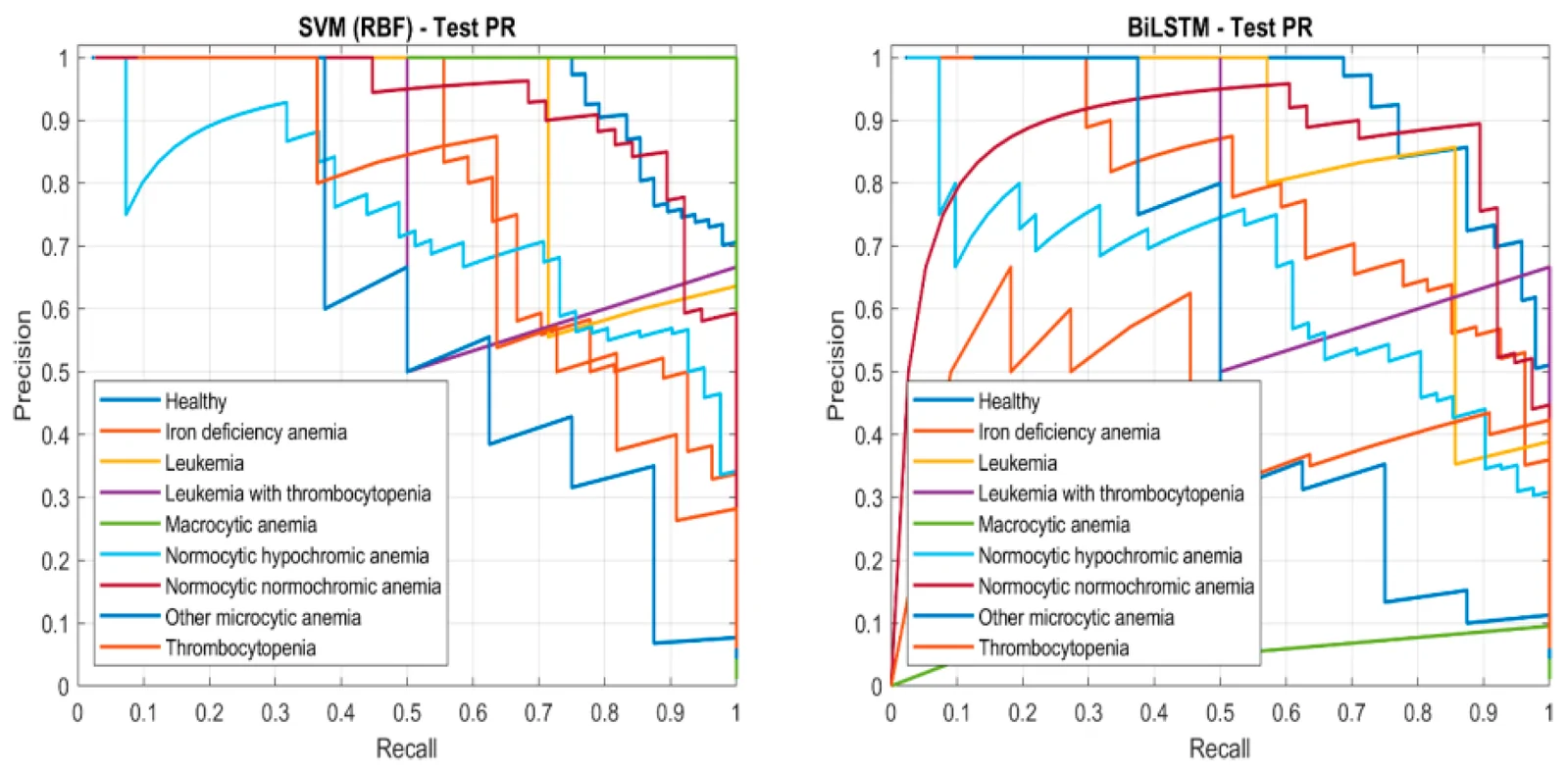
PR curves for selected baseline models and ALSS-Net on the test set.

Figure 5 and Figure 6 visualize the ROC and PR curves to examine the performance of the ALSS-Net architecture and the selected baseline models (MLP, SVM, BiLSTM) on the test dataset. Upon examining the ROC curves in Figure 5, it was observed that ALSS-Net formed the boundaries closest to the top-left corner, achieving a minimum class-wise AUC of 0.969. Furthermore, while the MLP emerged as a strong alternative, the SVM and, in particular, the BiLSTM models experienced significant performance losses in distinguishing certain classes. When evaluating the PR curves in Figure 6, which examine the models’ true robustness against class imbalances, the differentiation between the architectures became much clearer. ALSS-Net achieved balanced results in precision even at increasing recall levels and emerged as the top-performing model in the upper-right quadrant. It was observed that the MLP experienced early declines in some classes, while the SVM and BiLSTM models were unable to control false predictions as recall increased, resulting in sharp drops along the precision axis. These findings confirm that ALSS-Net not only delivered overall classification performance but also demonstrated a more balanced, robust, and reliable structure compared to its competitors in detecting each individual class.

**Figure 7:**
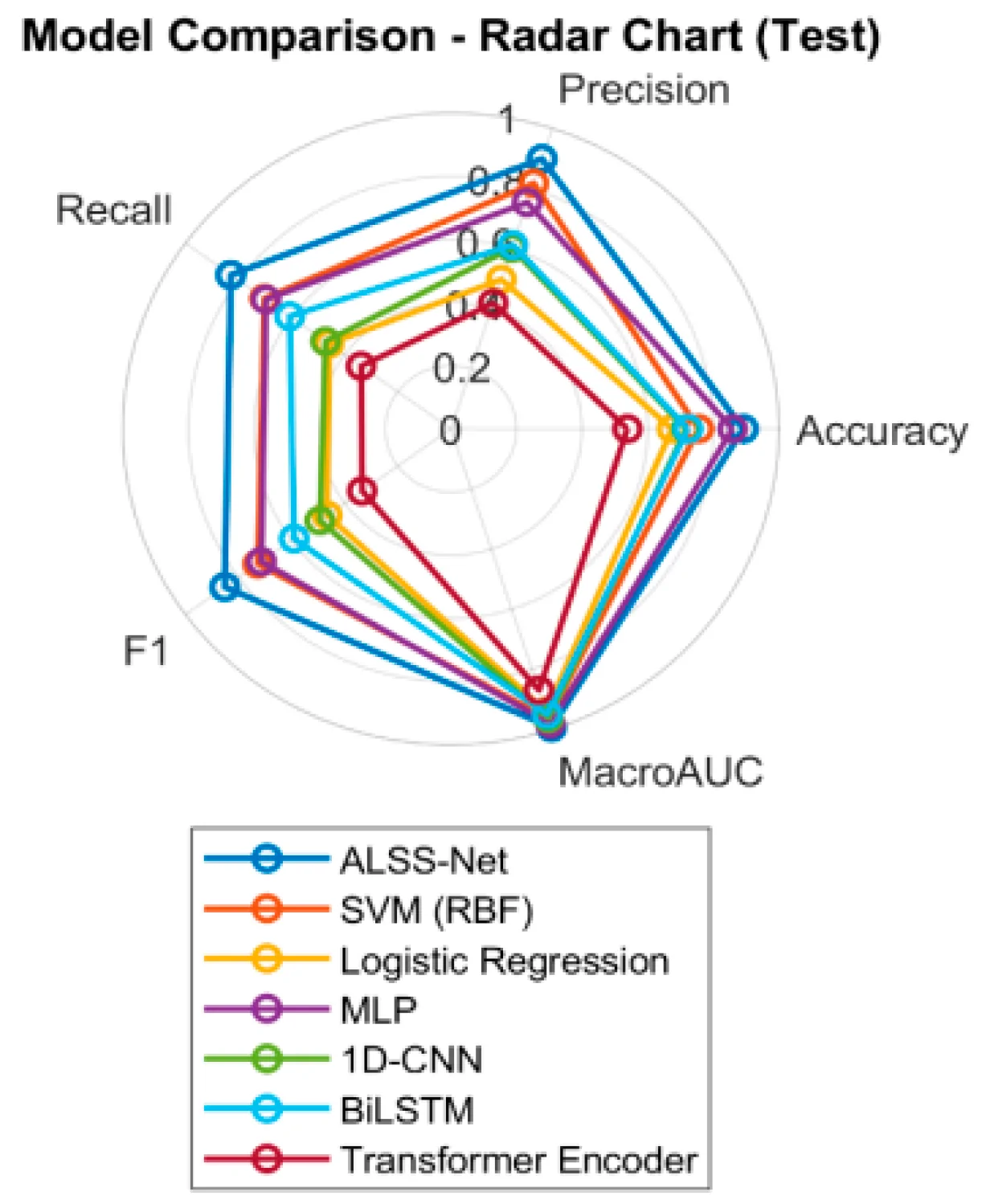
Radar chart comparing multi-metric classification performance across all evaluated models on the test set.

**Figure 8:**
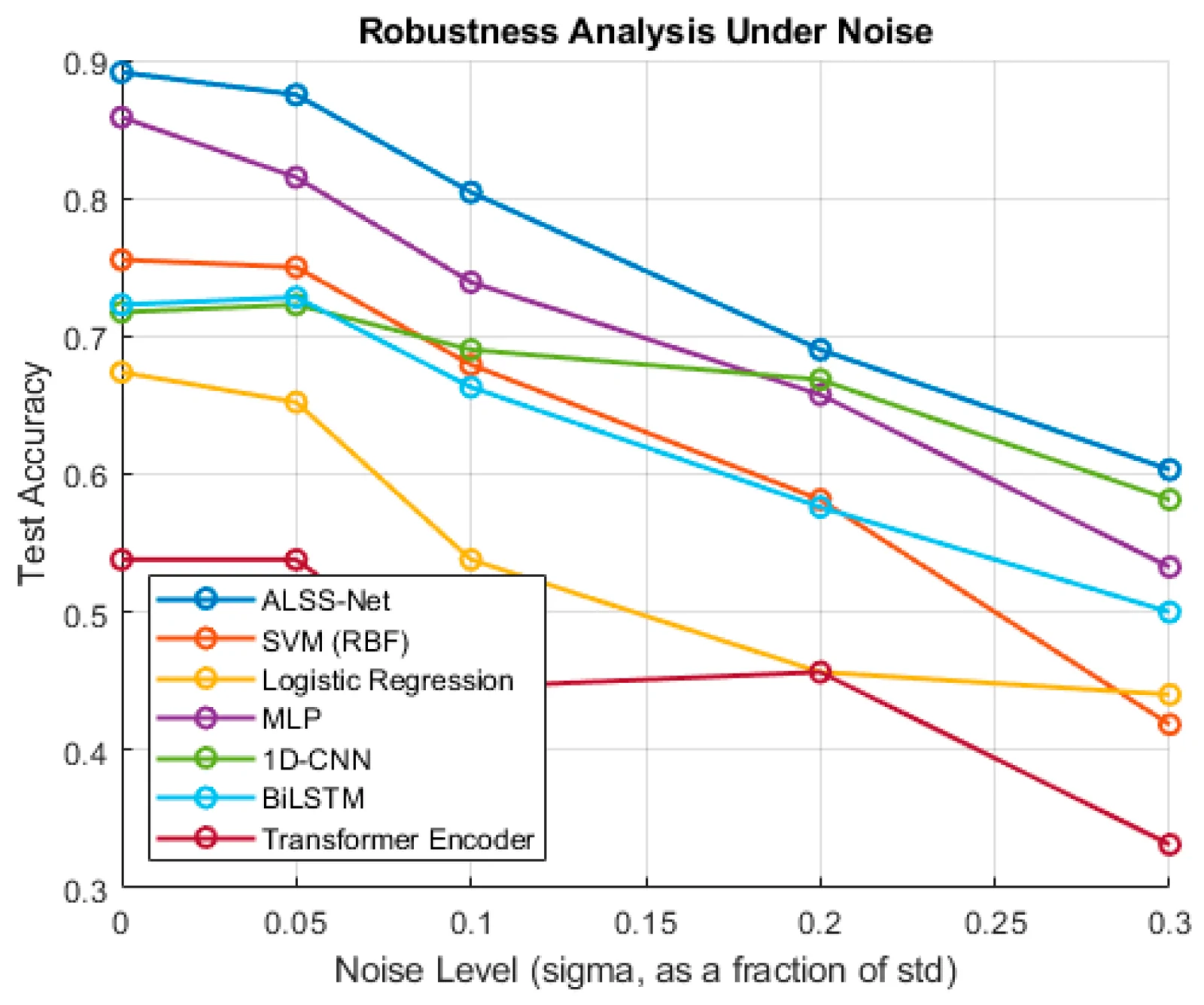
Robustness analysis comparing test accuracy degradation across models under increasing feature noise levels.

Figure 7 and Figure 8 analyze the multi-metric performance balance and stability of the proposed ALSS-Net architecture under noisy data in comparison with baseline models. Upon examining the radar chart in Figure 7, which simultaneously visualizes five different classification metrics, it was observed that ALSS-Net formed the outermost polygon on all axes, giving it an advantage over its competitors. MLP, its strongest alternative, covered a wide area on the Macro AUC and accuracy axes but lagged behind, narrowing toward the center, particularly for the F1 score and recall. To evaluate the validity of model performance under challenging conditions, the robustness analysis shown in Figure 8, obtained by adding increasing levels of feature noise (ranging from 0 to 0.3) to the test data, was examined in detail. In this context, the structural differences between the architectures became more pronounced. It was noticeable that the MLP model, which closely followed ALSS-Net on clean data, showed a decline in the graph as the noise level increased. The 1D-CNN model, which initially had a relatively low performance of approximately 72%, exhibited the flattest and most resilient curve against increasing noise. The fact that it outperformed strong competitors such as MLP, SVM, and BiLSTM at these noise levels provides evidence of the noise-suppression capacity of convolutional filters. The Transformer model’s accuracy dropped to approximately 33% when the data used contained high levels of noise. ALSS-Net stood out as both the most accurate and the most robust model against disruptive effects, achieving the highest accuracy even at the highest noise levels.

**Figure 9:**
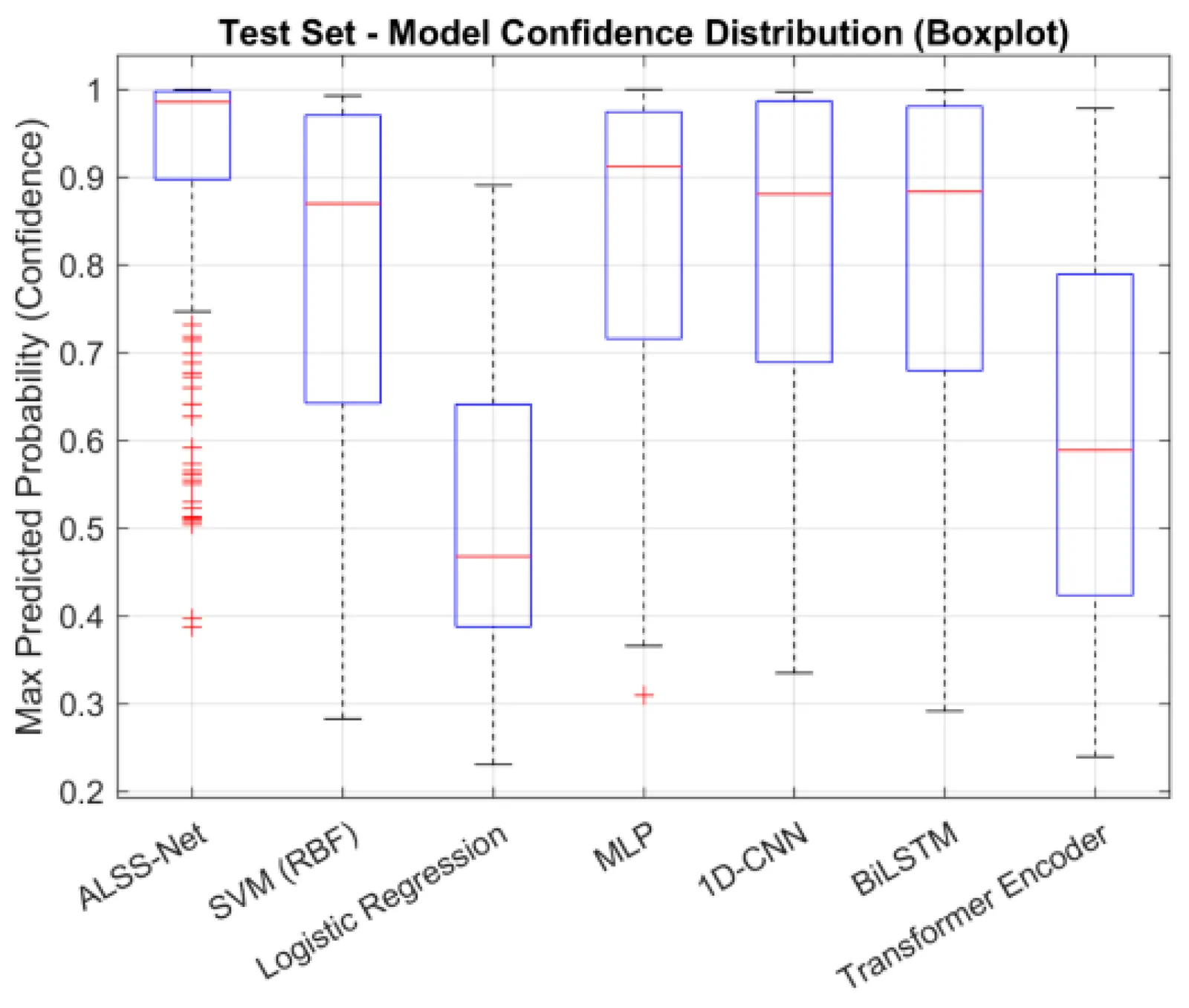
Boxplot distributions of maximum predicted probability (model confidence) for ALSS-Net and baseline models on the test set.

Figure 9 uses boxplots to visualize the maximum prediction probabilities of ALSS-Net and the baseline models on the test dataset. Upon examining the distributions, it was observed that ALSS-Net’s median value was close to the 1.0 line and that its interquartile range was tightly confined between 0.9 and 1.0. This distribution confirms that the vast majority of ALSS-Net’s classification decisions were made with a high level of confidence and that model calibration is stronger than that of its competitors. The red outliers indicate that the model experienced uncertainty only in a few specific and challenging data points. While the median values for architectures such as the MLP model, which came the closest to ALSS-Net in accuracy metrics, as well as SVM (RBF), 1D-CNN, and BiLSTM, appeared relatively high in the range of 0.85–0.91, it was noted that their box widths were spread over a wider range. This indicates that these baseline models experience structural fluctuations in their prediction probabilities and are not as stable in their decisions as ALSS-Net. The Logistic Regression and Transformer Encoder models, on the other hand, achieved low median values in the range of 0.40–0.60.

Table 7 compares the model calibration metrics and shows that the proposed ALSS-Net model outperforms its competitors in terms of probabilistic calibration and reliability on both the validation and test sets. ALSS-Net achieved a Brier score of 0.100 and an ECE value of 0.051 on the validation set, and a Brier score of 0.155 and an ECE value of 0.020 on the test set. Among the baseline models, the closest competitor, MLP, achieved a Brier score of 0.170 and an ECE of 0.071 on the validation set, and a Brier score of 0.218 and an ECE of 0.052 on the test set. While the SVM (RBF) and 1D-CNN models provided moderate calibration performance, the Logistic Regression and Transformer Encoder models lagged behind in probabilistic calibration, particularly due to their high Brier scores. The practical meaning of this difference is that a model with a lower calibration error reports confidence values that correspond more closely to observed accuracy, so that predictions issued at a confidence of approximately 0.90 are correct in approximately 90% of cases. This property is relevant to a decision-support setting, in which a clinician may reasonably choose to examine low-confidence predictions more carefully. Calibration alone, however, does not establish clinical safety. It is a statement about the aggregate agreement between confidence and accuracy on this particular test partition, and it does not indicate whether the errors that remain are clinically tolerable, how the model behaves on patient populations not represented in this dataset, or how the reported probabilities would be integrated into a clinical workflow. These questions require prospective evaluation on external data.

**Figure 10:**
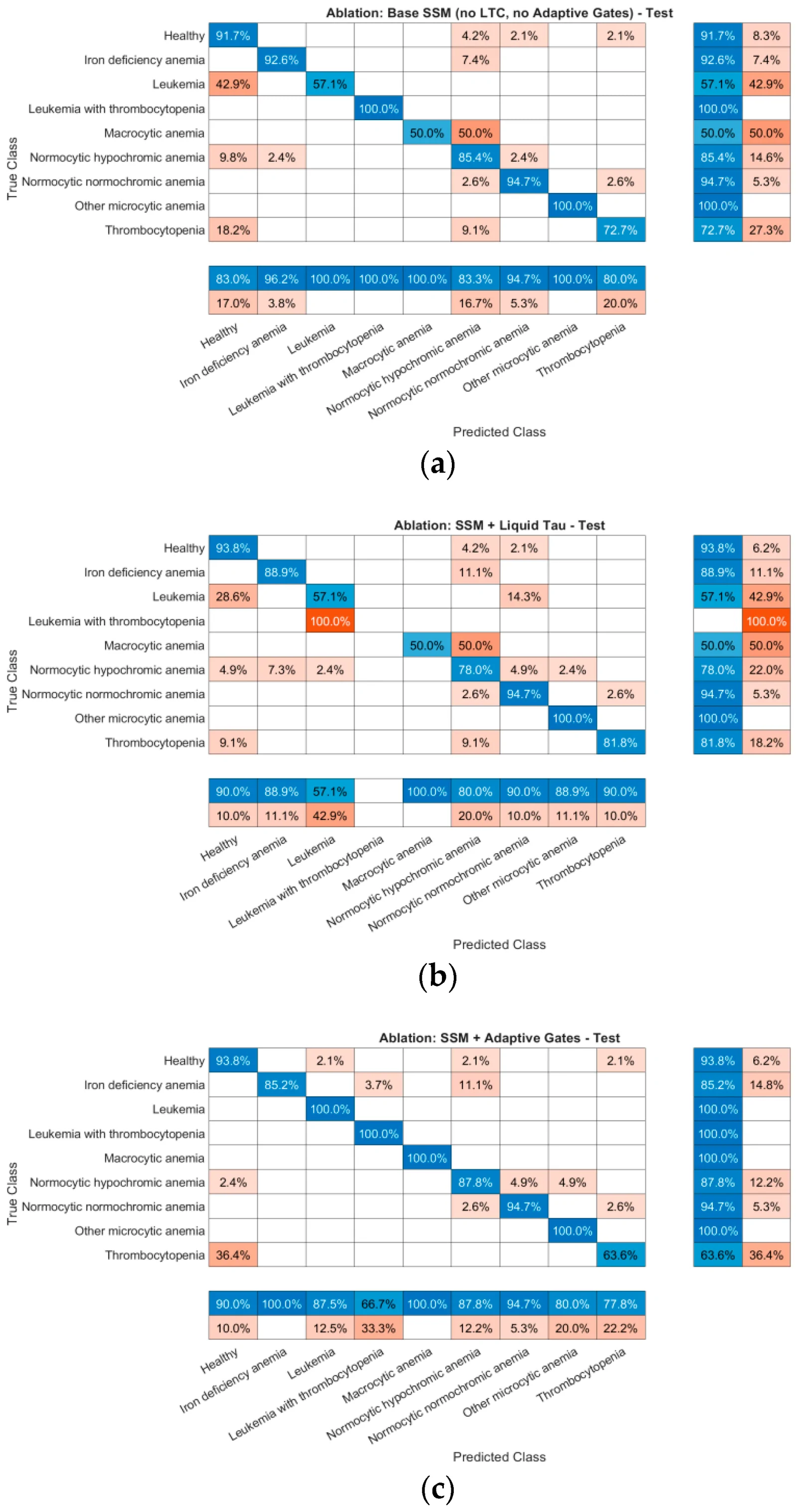
Row-normalized confusion matrices on the test set for ablation variants: (**a**) Base SSM, (**b**) SSM + Liquid Tau, and (**c**) SSM + Adaptive Gates.

Figure 10 illustrates row-normalized confusion matrices from ablation studies conducted on the test set to examine the contributions of ALSS-Net’s core components and the gradual changes in performance. This analysis, which includes three different configurations of ALSS-Net, examines the class-wise performance of the following variants: (a) Base SSM, stripped of LTC and adaptive gate mechanisms; (b) SSM + Liquid Tau, in which only LTC is integrated into the base SSM architecture; and (c) SSM + Adaptive Gates, in which adaptive gates are added to the base architecture.

In a detailed examination of the matrices, it was observed that the Base SSM model shown in Figure 10a was particularly inaccurate in classes such as Leukemia (57.1%) and Macrocytic anemia (50.0%). The SSM + Liquid Tau variant shown in Figure 10b, which incorporates LTC into the model architecture, increased accuracy to 81.8% in the Thrombocytopenia class while leaving the performance for Leukemia unchanged. The SSM + Adaptive Gates model shown in Figure 10c, which incorporates adaptive gates into the architecture, increased accuracy to 100.0% in the Leukemia and Macrocytic anemia classes, highlighting the role of this component in distinguishing challenging classes.

In addition, when changes in other classes were examined, differences in the mechanisms of action of the architectural variants became clearly evident. For example, the accuracy rate for the Normocytic hypochromic anemia class was 85.4% for variant (a), dropped to 78.0% for variant (b), and reached 87.8% for variant (c). The “Healthy” class achieved accuracy rates of 91.7%, 93.8%, and 93.8%, respectively, across all configurations. On the other hand, the “Thrombocytopenia” class dropped to 63.6% when adaptive gates were added, indicating a different interaction pattern in this subtype. Similarly, the accuracy for Iron deficiency anemia decreased from 92.6% to 85.2%. These findings indicate that each module has selective weights in representing and optimizing different features.

**Figure 11:**
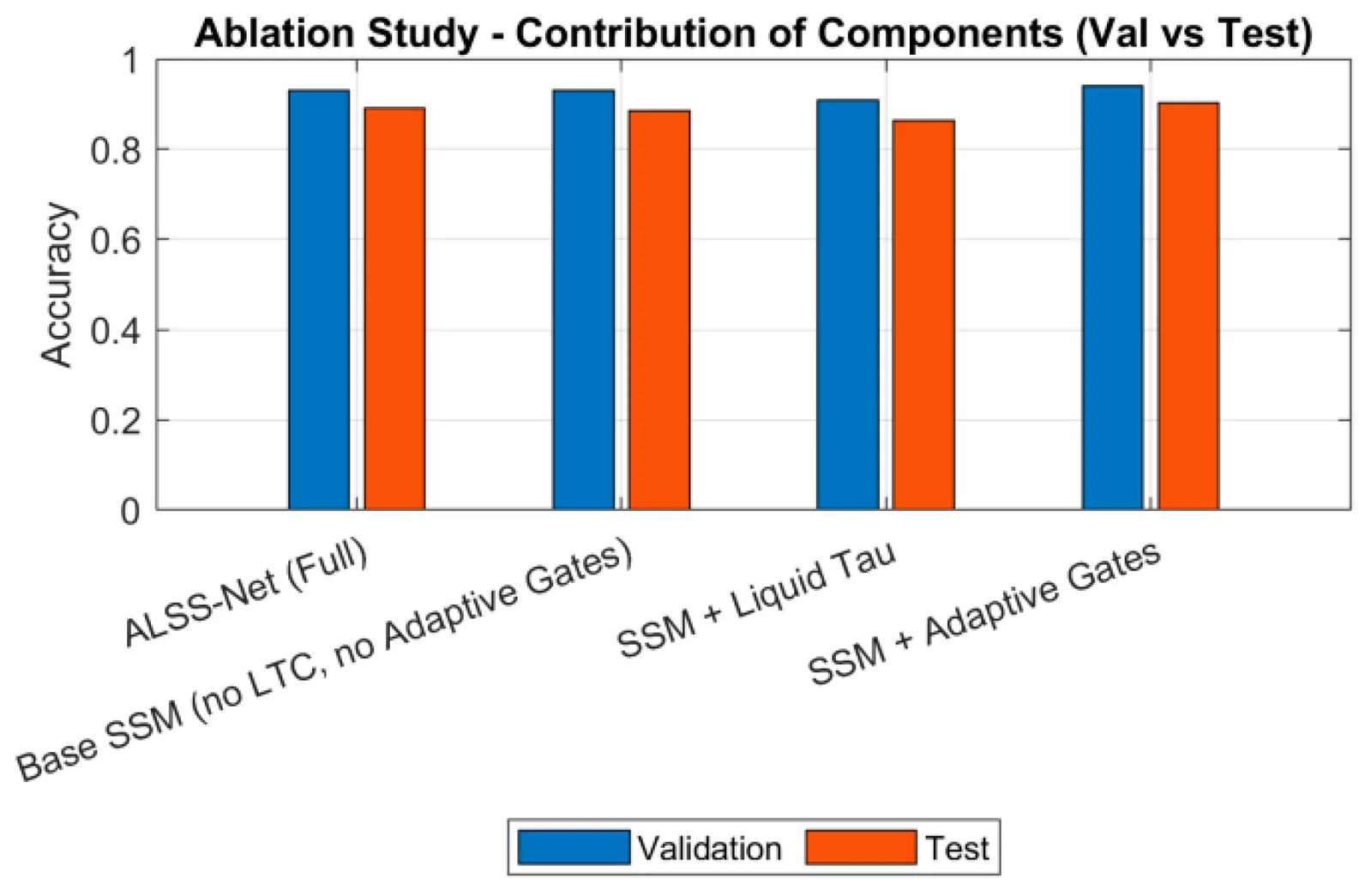
Validation and test accuracy comparison across ablation study variants highlighting the contribution of individual architectural components.

Figure 11 highlights the contribution of individual architectural components by showing a comparison of validation and test accuracy across ablation study variants. When examining the effects of individual architectural modules, the SSM + Liquid Tau variant exhibited limited changes in performance, while the SSM + Adaptive Gates variant achieved high accuracy rates. The fully equipped ALSS-Net architecture, on the other hand, successfully integrated all these components. Accordingly, the test results for the full architecture remained highly competitive compared to the model incorporating adaptive gates, clearly showing that the model possesses a robust generalization capability. Furthermore, the proposed architecture yielded improved results over the baseline SSM structure, outperforming the Base SSM model by approximately 0.58% on the test set. It should be noted, however, that the differences among the ablation variants on the test set are small relative to the width of the bootstrap confidence intervals reported in Table 6, and that the SSM + Adaptive Gates variant remains competitive with the complete model on this partition. The ablation therefore identifies adaptive gating as the component contributing most to the separation of the difficult classes, whereas the additional contribution of the liquid time-constant controller is better described as a stabilizing one, visible in the class-wise matrices of Figure 10 rather than in a large change in overall accuracy.

**Figure 12:**
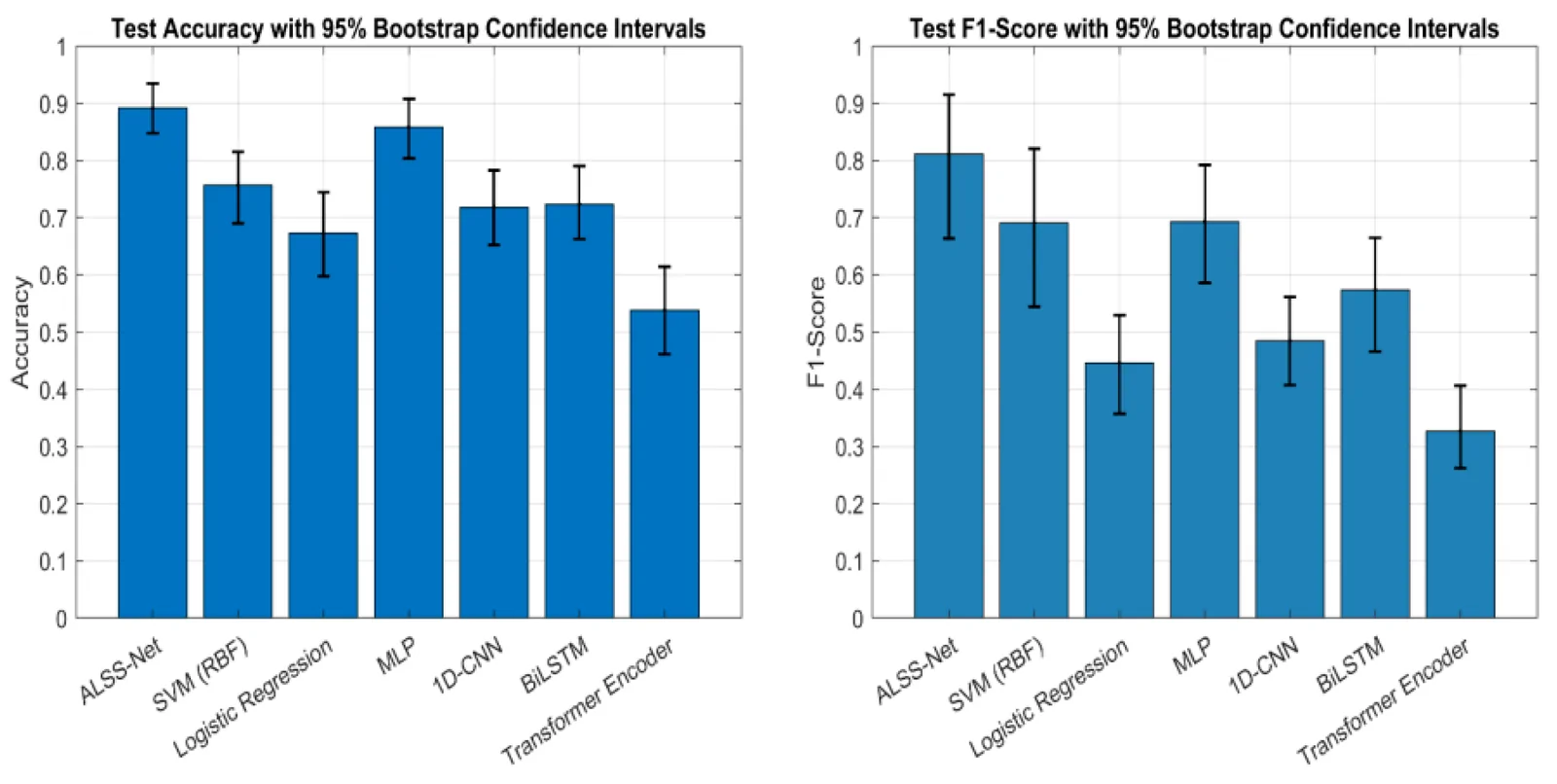
Test accuracy and F1-score bar charts with 95% bootstrap confidence intervals across all evaluated models.

Figure 12 presents a comparative analysis of the accuracy and F1-score performance of all evaluated models on the test set, along with their 95% bootstrap confidence intervals. Upon examining the graphs, it was observed that the proposed ALSS-Net architecture achieved the highest test accuracy, outperforming all other machine learning and deep learning models. The narrow confidence interval in the model’s accuracy plot indicates that the predictions are statistically stable and reliable. In addition to the high accuracy rate, ALSS-Net’s F1 score also ranked higher than that of its competitors in the performance evaluation. Although its F1-score value was numerically slightly lower than its accuracy rate, ALSS-Net maintained a clear superiority over all baseline models across both metrics. When evaluating the performance of other models, the MLP model was found to be the most competitive in both metrics. On the other hand, the Transformer Encoder and Logistic Regression models exhibited the weakest performance in terms of both accuracy and F1-score. They also showed a more inconsistent structure with wide confidence intervals. Overall, these results underscore that ALSS-Net possesses a consistent generalization capability.

## 4. Discussion

This section presents pairwise statistical significance tests (*p*-values) and performance comparisons among all the models evaluated.

Table 8 shows the detailed results of the pairwise statistical significance tests and performance comparisons conducted among all evaluated models. Using the generally accepted *p* < 0.05 threshold in statistical analyses as a reference, this table explains whether the performance differences between the models are random or statistically significant. A comprehensive review of the table data reveals that, when compared to the SVM (RBF), Logistic Regression, 1D-CNN, BiLSTM, and Transformer Encoder models, the ALSS-Net architecture provided statistically significant superiority with a *p*-value of < 0.001. This finding confirms, through statistical tests, that ALSS-Net’s high performance relative to other models is not due to chance. One of the most notable statistical findings in the table comes from the comparison between ALSS-Net and MLP, the two best-performing models tested. The *p*-value for this comparison was calculated as 0.2636. Even though ALSS-Net yields higher results based on general metrics, the performance difference between ALSS-Net and MLP is not statistically significant. On the other hand, the MLP model was determined to be the second-best alternative by establishing a statistically significant superiority at the *p* < 0.001 level against all competitors other than ALSS-Net. Analysis of traditional machine learning algorithms and basic deep learning models revealed that they form statistically equivalent sets. In particular, the comparison between the 1D-CNN and BiLSTM models yielded a *p*-value of 1.000. Similarly, no significant performance difference was found between the traditional SVM (RBF) model and the deep learning-based 1D-CNN (*p* = 0.3487) and BiLSTM (*p* = 0.4047) models. This indicates that models processing spatial and temporal features in different ways form an intermediate group in terms of performance. The Transformer Encoder, on the other hand, exhibited limited efficacy on this dataset, yielding statistically significant differences in all pairwise comparisons with the other architectures. In conclusion, the pairwise comparison tests presented in Table 8 confirm that the ALSS-Net and MLP models were significantly more successful than all other approaches in distinguishing the classes in the dataset. The statistical validity of the success achieved by the proposed ALSS-Net is thus highlighted.

Taken together, these evaluations indicate that the proposed ALSS-Net architecture performs favourably across the range of criteria examined on this dataset. It obtained the highest accuracy among the models compared; this advantage was statistically significant against five of the six baselines, while the difference with respect to the MLP baseline was not (Table 8). Furthermore, ALSS-Net’s superiority is not limited to its basic classification performance. The Brier score, which is of critical importance in clinical decision support systems, along with ECE and reliability diagrams, has shown that the model’s predictions exhibit a high degree of stability. Supported by boxplot analyses, this finding reveals that the model stands apart from traditional deep learning architectures, which tend to make even incorrect predictions with high confidence. The architecture’s behavior under challenging conditions and the contributions of its structural components are also particularly compelling. Ablation studies show that the integration of the LTC and adaptive gate mechanisms in the model plays a complementary and balancing role, particularly in the separation of challenging classes. Furthermore, its success in the face of increasing feature noise and its resilience to class imbalances in the PR curves confirm that ALSS-Net offers a satisfactory architecture. On the other hand, it is noteworthy that the MLP model, which stands out as the most competitive alternative, experiences rapid breakdowns under noise despite its success on clean data. Moreover, the wider confidence intervals observed for the MLP model highlight the dynamic architectural advantage provided by ALSS-Net. In summary, this section shows that ALSS-Net is not merely a simple classifier that offers high accuracy rates, but also a reliable deep learning framework with stable training dynamics, statistical validity, and resilience to environmental noise.

## 5. Conclusions

In this study, ALSS-Net was proposed to classify difficult-to-diagnose types of anemia with high accuracy, robustness, and statistical reliability by analyzing complex tabular data. The model developed for this purpose was comprehensively benchmarked against six baseline models using multidimensional performance metrics. By integrating core SSM, LTC, and Adaptive Gate mechanisms into a unified framework, this dynamic architecture was able to overcome the inherent limitations faced by traditional machine learning algorithms and standard deep learning models. Experimental results demonstrated that ALSS-Net achieved a test accuracy of 89.13% and an F1-score of 0.8513, the highest values among the models compared (SVM, Logistic Regression, MLP, 1D-CNN, BiLSTM, and Transformer Encoder). These differences were assessed using McNemar’s test and bootstrap confidence intervals. The advantage over five of the six baselines was statistically significant (*p* < 0.001), whereas the difference with respect to the MLP baseline was not (*p* = 0.2636). The evidence therefore supports a clear separation from the weaker baselines, together with an advantage over the strongest baseline that is consistent across metrics but is not statistically established on a test partition of this size. Although the MLP model emerged as the most competitive alternative in terms of overall metrics, ALSS-Net’s ability to recover cases in minority and complex subclasses in which most baselines failed entirely indicates more balanced behaviour under class imbalance on this partition; as noted below, the small number of test cases in those classes limits how far this observation can be extended.

Beyond its classification performance, one of ALSS-Net’s most notable advantages lies in its probabilistic calibration and noise tolerance. By achieving the lowest Brier score and ECE values in calibration analyses, the model yielded reliable probabilistic outputs that were well aligned with the observed outcomes. At the same time, its resilience to increasing feature noise indicates that the architecture degrades gracefully under simulated measurement error, which is a desirable property for laboratory data, although this behaviour was assessed through synthetic perturbation rather than through observed analytical variability. Detailed ablation analyses have also confirmed that the architecture’s overall stability stems from the cumulative contribution of the integrated time-constant and gate modules. Ultimately, ALSS-Net is not merely an algorithm that delivers high metric scores for medical analysis processes. It is also an artificial intelligence framework that provides reliable probabilistic outputs, is robust against noise distortions, and has statistical validity. Despite these promising results, the ALSS-Net architecture has several limitations. A key limitation is that running mechanisms such as SSM and LTC on static tabular datasets that lack temporal dependency, such as anemia data, may limit the architecture’s full dynamic potential. In addition, adaptive gates and continuous-time parameter updates may increase computational complexity compared to traditional models. Finally, the black-box nature of this multi-component architecture may limit model explainability, which is of critical importance for medical diagnostic applications, thereby making it difficult for physicians to interpret the reasoning behind classification decisions.

Several of these limitations bear directly on the strength of the claims that can be made. All results were obtained on a single open-access dataset of 1232 records and evaluated on one test partition of 184 samples, without external validation on data originating from another institution, analyzer, or acquisition protocol. The study therefore establishes computational classification performance under a fixed experimental protocol, and it does not establish clinical utility. The rarest diagnostic categories are represented by very few test cases; so, the class-wise scores reported for those categories carry substantial uncertainty and should not be read as evidence of generalization to those conditions. The provenance limitations of the source dataset described in Section 2, in particular the absence of documented labeling criteria and of patient-level metadata, further restrict the conclusions that can be drawn about the populations to which these results would transfer. A further limitation concerns the comparison itself: as described in Section 2.2.4, the configurations of ALSS-Net and of the baseline models were fixed a priori as conventional settings for each model family and were not obtained by a hyperparameter search, and the neural baselines were trained under a smaller maximum epoch budget and a constant rather than cosine-annealed learning rate. The reported differences therefore hold between these particular configurations under a common protocol, and a systematic search could alter the relative ordering of the models. Accordingly, ALSS-Net is presented here as a methodological contribution and as a candidate component for future decision-support research, and not as a system ready for diagnostic deployment.

Two further restrictions concern what the dataset does not contain. As noted in Section 1, the hemoglobin thresholds by which anemia is defined are set with reference to age, sex, and pregnancy status, yet none of these variables is distributed with the records; so, the models operate on the complete-blood-count panel alone and cannot apply the demographic adjustment that clinical interpretation would ordinarily involve. The absence of iron studies places a comparable limit on the “iron deficiency anemia” category, and the absence of smear, blast, and immunophenotyping data on the leukemia categories, as set out in Section 2. External validation is therefore a necessary next step rather than an optional extension: complete-blood-count parameters vary with analyzer platform, calibration, and reference population, and performance measured on a single source cannot be assumed to transfer to another. We regard evaluation on data from other institutions and analyzers, together with labels established under a documented clinical reference standard, as a prerequisite for any claim of clinical usefulness.

With respect to explainability, the present study evaluates predictive performance, calibration, and robustness rather than clinical interpretability, and no attribution experiment was carried out. Several approaches could nevertheless be applied to ALSS-Net without modifying the architecture. Model-agnostic methods such as permutation feature importance and SHAP are directly applicable, since the model consumes a fixed-length vector of named clinical variables. Gradient-based attribution such as integrated gradients is also applicable, because the forward pass is differentiable end to end. In addition, the architecture provides an internal handle that generic methods do not: the quantities τ_d_, Δ_d_, B_d_, and C_d_ are generated per feature token and per sample, so that the learned time constants indicate how persistently each clinical variable influences the accumulated representation, while the gate magnitudes indicate how strongly that variable is written into and read out of the state for an individual patient. Examining these quantities across patients and comparing them with established hematological reasoning is a natural route to establishing whether the internal behaviour of the model is clinically coherent, and it is left to future work.

Future work aims to investigate the adaptation of this dynamic architecture on larger-scale clinical datasets encompassing different disease modalities and to perform optimizations for its integration into real-time medical decision support systems.

## Figures and Tables

**Table 1:** Comprehensive ALSS-Net Framework for Tabular Classification.

**Input:** Raw data table Data (last column = class label), configuration cfg **Output:** Trained models, evaluation tables, figures, computational and statistical analyses
1: Initialize the experimental configuration and random seed 2: Remove duplicate samples, verify that no missing values remain, and extract predictors X and class labels Y 3: Split (X, Y) into stratified Training, Validation, and Test subsets (70/15/15; *n* = 863/185/184) 4: Normalize features using z-score statistics computed from the training set and one-hot encode class labels 5: Train the proposed ALSS-Net using the Training and Validation sets with Adam optimization and early stopping; retain the best-performing model according to the validation set and save the trained model 6: Train baseline models {SVM, Logistic Regression, MLP, 1D-CNN, BiLSTM, Transformer} using the same data partitions 7: Evaluate each model on the Validation and Test sets by computing Accuracy, Precision, Recall, Specificity, F1-score, Macro-AUC, confusion matrices, per-class performance metrics, ROC curves, Precision–Recall curves, and reliability diagrams 8: Compare the proposed ALSS-Net with all baseline models using quantitative metrics, bar charts, radar plots, heatmaps, and pairwise McNemar significance tests 9: Perform an ablation study by retraining ALSS-Net with the LTC controller and/or adaptive gating mechanism disabled, and compare all variants with the full model 10: Measure computational efficiency by recording training time, inference time per sample, throughput (FPS), and parameter count 11: Assess robustness by progressively injecting Gaussian noise into the test data and re-evaluating all models 12: Perform bootstrap statistical analysis to estimate 95% confidence intervals for Accuracy and F1-score and statistically compare ALSS-Net with each baseline model. 13: Assess model generalization by comparing Training and Validation performance 14: Export all trained models, evaluation tables, figures, and statistical results 15: Return trained models, evaluation tables, ablation results, computational efficiency analysis, robustness analysis, and statistical results

**Table 2:** Training configuration and optimization hyperparameters for ALSS-Net.

Hyperparameter	Value
Optimizer	Adam
Initial learning rate	1 × 10^−3^
Learning rate schedule	Cosine annealing
Adam β_1_ (gradient decay)	0.90
Adam β_2_ (squared gradient decay)	0.999
Batch size	32
Maximum epochs	150
Early stopping patience	20 epochs (monitored on validation loss)
Gradient clipping (global norm)	5.0
L2 weight regularization (λ)	1 × 10^−4^
Dropout rate	0.20
Loss function	Categorical cross-entropy + L2 penalty
Random seed	10
Train/Validation/Test split	70%/15%/15% (863/185/184 samples)

**Table 3:** Architectural variants and component configurations evaluated in the ablation study.

Variant	LTC Controller	Adaptive Gates (B/C)	Description
Base SSM	✗	✗	A static state-space baseline with fixed time-constant and fixed (unit) input/output gates; reveals the contribution of the core recurrent structure alone.
SSM + Liquid Tau	✓	✗	Adds the liquid time-constant controller (adaptive τ, Δ) on top of the base SSM, while input/output gates remain fixed; measures the effect of adaptive temporal dynamics.
SSM + Adaptive Gates	✗	✓	Adds the learnable B/C gating mechanism on top of the base SSM, while τ and Δ remain fixed constants; highlights the effect of adaptive gating alone.
ALSS-Net	✓	✓	The complete proposed architecture combining both the liquid time-constant controller and adaptive gating.

“✗”: No and “✓”: Yes.

**Table 4:** Performance comparison of ALSS-Net and baseline models on the validation set.

Model (Val.)	Accuracy	Precision	Recall	F1	AUC
ALSS-Net	0.9297	0.8104	0.8051	0.8013	0.9977
SVM (RBF)	0.8270	0.7708	0.7809	0.7464	0.9806
Logistic Regression	0.7243	0.4741	0.4409	0.4450	0.9454
MLP	0.8810	0.7933	0.7450	0.7425	0.9897
1D-CNN	0.7459	0.6321	0.5048	0.5189	0.9768
BiLSTM	0.7459	0.5378	0.5058	0.5130	0.9602
Transformer Encoder	0.5405	0.3943	0.3206	0.3136	0.8857

**Table 5:** Performance comparison of ALSS-Net and baseline models on the test set.

Model (Test)	Accuracy	Precision	Recall	F1	AUC
ALSS-Net	0.8913	0.8982	0.8288	0.8513	0.9931
SVM (RBF)	0.7554	0.8170	0.7020	0.7270	0.9649
Logistic Regression	0.6739	0.4972	0.4583	0.4654	0.9403
MLP	0.8586	0.7533	0.6960	0.7137	0.9805
1D-CNN	0.7173	0.6013	0.4718	0.4933	0.9619
BiLSTM	0.7228	0.6117	0.6070	0.5906	0.9495
Transformer Encoder	0.5380	0.4180	0.3382	0.3340	0.8689

**Table 6:** Bootstrap statistical confidence intervals (95%) and standard deviations for Accuracy and F1-score across models on the test set.

Model (Test)	Accuracy CI Low	Accuracy CI High	Accuracy Std	F1 CI Low	F1 CI High	F1 Std
ALSS-Net	0.8478	0.9347	0.0227	0.6635	0.9146	0.0632
SVM (RBF)	0.6902	0.8152	0.0320	0.5441	0.8201	0.0692
Logistic Regression	0.5978	0.7445	0.0360	0.3569	0.5297	0.0505
MLP	0.8043	0.9076	0.0264	0.5864	0.7927	0.0561
1D-CNN	0.6521	0.7826	0.0328	0.4074	0.5607	0.0386
BiLSTM	0.6630	0.7907	0.0334	0.4664	0.6644	0.0521
Transformer Encoder	0.4619	0.6141	0.0368	0.2619	0.4066	0.0373

**Table 7:** Model calibration metrics including Brier score and ECE on validation and test sets.

Model	Brier Score Val	ECE Val	Brier Score Test	ECE Test
ALSS-Net	0.100	0.051	0.155	0.020
SVM (RBF)	0.248	0.064	0.342	0.054
Logistic Regression	0.473	0.226	0.507	0.163
MLP	0.170	0.071	0.218	0.052
1D-CNN	0.342	0.061	0.360	0.102
BiLSTM	0.331	0.075	0.404	0.125
Transformer Encoder	0.593	0.091	0.579	0.115

**Table 8:** Pairwise statistical significance testing (*p*-values) and performance comparison across all evaluated models.

Model A	Model B	*p* Value	Significant
ALSS-Net	SVM (RBF)	<0.001	✓
ALSS-Net	Logistic Regression	<0.001	✓
ALSS-Net	MLP	0.2636	✗
ALSS-Net	1D-CNN	<0.001	✓
ALSS-Net	BiLSTM	<0.001	✓
ALSS-Net	Transformer Encoder	<0.001	✓
SVM (RBF)	Logistic Regression	0.0455	✓
SVM (RBF)	MLP	<0.001	✓
SVM (RBF)	1D-CNN	0.3487	✗
SVM (RBF)	BiLSTM	0.4047	✗
SVM (RBF)	Transformer Encoder	<0.001	✓
Logistic Regression	MLP	<0.001	✓
Logistic Regression	1D-CNN	0.3317	✗
Logistic Regression	BiLSTM	0.2718	✗
Logistic Regression	Transformer Encoder	0.0039	✓
MLP	1D-CNN	<0.001	✓
MLP	BiLSTM	<0.001	✓
MLP	Transformer Encoder	<0.001	✓
1D-CNN	BiLSTM	1.000	✗
1D-CNN	Transformer Encoder	<0.001	✓
BiLSTM	Transformer Encoder	<0.001	✓

“✗”: No and “✓”: Yes.

## Data Availability

The data that support the findings of this study are openly available in Kaggle at https://www.kaggle.com/datasets/ehababoelnaga/anemia-types-classification, reference number [33]. Access Time: 2 July 2026.
